# Quantum engineering of non-equilibrium efficient p-doping in ultra-wide band-gap nitrides

**DOI:** 10.1038/s41377-021-00503-y

**Published:** 2021-03-31

**Authors:** Ke Jiang, Xiaojuan Sun, Zhiming Shi, Hang Zang, Jianwei Ben, Hui-Xiong Deng, Dabing Li

**Affiliations:** 1 State Key Laboratory of Luminescence and Applications, Changchun Institute of Optics, Fine Mechanics and Physics, Chinese Academy of Sciences, Dongnanhu Road No. 3888, Changchun, 130033 China; 2 Center of Materials Science and Optoelectronics Engineering, University of Chinese Academy of Sciences, Yuquan Road No. 19, Beijing, 100049 China; 3 State Key Laboratory of Superlattices and Microstructures, Institute of Semiconductors, Chinese Academy of Sciences, Qinghuadong Road No. 35, Beijing, 100083 China

**Keywords:** Optical materials and structures, Electronics, photonics and device physics

## Abstract

Ultra-wide band-gap nitrides have huge potential in micro- and optoelectronics due to their tunable wide band-gap, high breakdown field and energy density, excellent chemical and thermal stability. However, their application has been severely hindered by the low p-doping efficiency, which is ascribed to the ultrahigh acceptor activation energy originated from the low valance band maximum. Here, a valance band modulation mode is proposed and a quantum engineering doping method is conducted to achieve high-efficient p-type ultra-wide band-gap nitrides, in which GaN quantum-dots are buried in nitride matrix to produce a new band edge and thus to tune the dopant activation energy. By non-equilibrium doping techniques, quantum engineering doped AlGaN:Mg with Al content of 60% is successfully fabricated. The Mg activation energy has been reduced to about 21 meV, and the hole concentration reaches higher than 10^18^ cm^−3^ at room temperature. Also, similar activation energies are obtained in AlGaN with other Al contents such as 50% and 70%, indicating the universality of the quantum engineering doping method. Moreover, deep-ultraviolet light-emission diodes are fabricated and the improved performance further demonstrates the validity and merit of the method. With the quantum material growth techniques developing, this method would be prevalently available and tremendously stimulate the promotion of ultra-wide band-gap semiconductor-based devices.

## Introduction

Ultra-wide band-gap (UWBG) nitrides, as a new generation of semiconductor, have been playing a central role in the fields of efficient deep-ultraviolet illumination and detection, high-frequency and high-power electronic devices, due to their tunable direct UWBG, high breakdown field, excellent chemical and thermal stability^1–3^. Moreover, recent advances on nitride-based semiconductor-superconductor and monolithic integrated optical communication chips further demonstrate the brilliant prospect of UWBG nitrides^4,5^. In practice, it is crucial to artificially tailor semiconductor properties by intentional doping to realize electron (n-type) or hole (p-type) conducting before they can be adopted to these sophisticated optoelectronics or microelectronics. However, for UWBG nitrides, some issues including dopant solubility, self-compensation, and high acceptor activation energy (*E*_a_), seriously hinder their efficient p-doping, gradually becoming the main obstacle in realizing high-performance devices^6^. The solubility of the most frequently used Mg dopant, which is the major p-type dopant in nitrides, decreases sharply as the Al content increases^6,7^. The formation energy (*E*_F_) of nitrogen vacancy (*V*_N_) is low and the *V*_N_ density sharply increases as Al content promotes, which naturally provides excess electrons to compensate holes, further resulting in low p-type conduction^8^. Nowadays, issues of solubility and self-compensation can be moderated by growth conditions^9,10^. Unfortunately, the high *E*_a_, which is indeed a physical limitation, is still hampering the p-doping efficiency. Hence, the control of *E*_a_ turns into the core issue.

Generally, *E*_a_ is determined by the alignment of dopant level with respect to the band edge (BE) in absolute energy scale^11,12^. A semiconductor is difficult to be p-type (n-type) doped if the valance band maximum (VBM) (conduction band minimum, CBM) is too low (high). For example, in wide band-gap semiconductors such as nitrides and oxides, there is a long-standing problem: asymmetric doping, namely they can be easily n-doped but cannot be well p-doped^13,14^. It mainly results from the very low VBM induced by the large electronegativity of anion. This problem becomes even more challenging in UWBG nitrides. As the Al content increases, the band-gap of UWBG nitrides can be tuned from 3.4 to 6.2 eV from GaN to AlN. The VBM of AlN is about 0.7–0.8 eV lower than that of GaN^15^. Consequently, the VBM decreases as the Al content increases in UWBG nitrides, resulting in extremely high acceptor *E*_a_ and low p-doping efficiency in Al-rich AlGaN alloys. This is indeed true in reality. For the most-frequently-used p-type dopant Mg in nitrides, the *E*_a_ increases from 200 meV in GaN to as high as 630 meV in AlN^13,16,17^, which are far greater than the thermal energy of 26 meV at room temperature (RT). Therefore, the hole is impossible to release efficiently in the UWBG nitrides. Although lots of approaches including delta-doping, superlattice doping, and polarization doping have been proposed to improve the p-doping efficiency of UWBG nitrides^17–20^, there are few reports on high-efficient p-doped AlGaN with Al content higher than 50% due to the underlying physical limitation.

From this point of view, once the BE or the dopant energy levels can be artificially tuned, the *E*_a_ consequently can be controlled. To reduce the acceptor *E*_a_ in UWBG nitrides, two ways can be attempted. One is to lower the acceptor level and the other is to lift the VBM up. As is known that the dopant energy level cannot be easily changed because the dopant effective Bohr radius is too small to be effectively confined, which is widely demonstrated by nanocrystalline doping^21,22^. Therefore, to tune the BE seems to be more feasible. For instance, the strong VB offset bowing of ZnO_1*−x*_S_*x*_, which results from the local Zn–S bonds, can enhance p-doping of ZnO alloys^23^. While, for UWBG nitrides, the BE almost linearly varies with the content, indicating the Ga–N and Al–N bonds can equally affect the BE, resulting in almost linear dependence of *E*_a_ on Al content^6,7^. It implies the less probability to tune the BE of random UWBG nitrides by local bonds. Fortunately, it is noticed that some core-shell structures like Si/Ge and InAs/GaAs nanowires are utilized to dope nanocrystalline and the carrier concentration is significantly improved^24,25^. The improvement is attributed to the reduction of *E*_a_ of the system, which comes from the BE shift induced by the embedded local quantum structures.

**Fig. 1:**
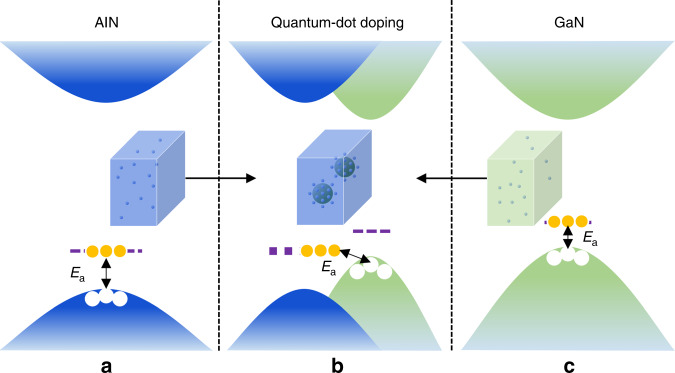
VB modulation mode to lower the acceptor *E*_a_ in UWBG nitrides. Acceptors are randomly doped in **a** AlN and **c** GaN. Both have high *E*_a_ in this condition. **b** GaN QDs are embedded in the AlN matrix and acceptors are doped in the AlN matrix and concentrate near the interface

The phenomenon that *E*_a_ can be tuned by quantum structures gives us inspiration to dope UWBG nitrides. In this work, we have theoretically proposed a VB modulation mode to achieve high-efficient p-type UWBG nitrides and have technically conducted a quantum engineering to realize the idea, in which GaN quantum-dots (QDs) are buried in UWBG nitride matrix to produce a new BE in the system and thus to tune the system *E*_a_. Figure 1 schematically depicts the acceptor *E*_a_ reduction mechanism of adopting QDs to UWBG nitrides to alter the BE of the system. When the acceptors are randomly doped in bulk UWBG nitrides, the acceptor level is so deep that few acceptors can ionize (Fig. 1a, c). In thermodynamic equilibrium growth conditions, no matter how to change the Al content, it is hard to make the acceptor *E*_a_ less than one hundred meV. However, it is noticed that the VBM of GaN is 0.7–0.8 eV higher than that of AlN^15^. If the GaN quantum structures can be locally formed in the UWBG nitride matrix, the actual VBM of the system would lift up to get closer to the acceptor level in the UWBG nitride matrix, resulting in lower acceptor *E*_a_ (Fig. 1b). Considering that Mg has a *E*_a_ of 630 meV in AlN and the VBM of AlN is 0.7–0.8 eV lower than that of GaN, an extreme low *E*_a_ can be obtained when GaN QDs are buried in AlN. Although the quantum-confinement effect (QCE) can shift down the VBM, a low *E*_a_ can also be attained by controlling the shape, size, and distribution of the embedded QDs.

## Results

**Fig. 2:**
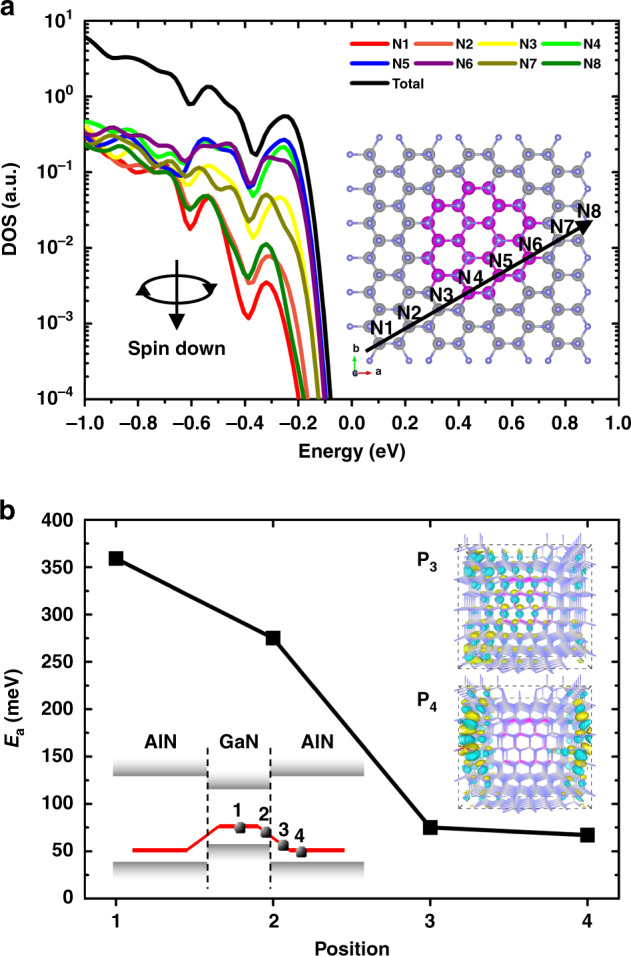
First-principle calculations. **a** Spin-down PDOS of the total and different N atoms in AlN:GaN QDs system. Because the spin-up PDOS distribution of un-doped systems are image symmetrical to the spin-down PDOS and the Mg doping mainly contribute to the spin-down PDOS, only spin-down PDOS distributions are displayed for simplicity. **b** *E*_a_ of Mg-doped AlN:GaN QDs system at different doping positions. The left inset illustrates the *E*_a_ variation with doping position and the right insets are the wavefunctions of the orbitals of Mg dopant near the VBM (spin-down) at positions 3 and 4

To theoretically confirm the VB modulation mode and the quantum engineering doping method, first-principle calculations are applied to estimate the *E*_a_ of the AlGaN system. We first calculate the band-gap of bulk GaN (AlN) and the *E*_a_ of bulk GaN:Mg (AlN:Mg) (Fig. S1). The resulted band-gap of bulk GaN (AlN) is 3.353 eV (5.749 eV), which is consistent with the reported values^26,27^. When Mg is doped in bulk GaN (AlN), the calculated *E*_a_ of bulk GaN:Mg (AlN:Mg) is 0.141 eV (0.515 eV), which also agrees with the experiments^28,29^. In the quantum engineering doping calculation, the model in which GaN QDs are embedded in the AlN matrix is established (Fig. S2). It is found that the effective band-gap of un-doped AlN:GaN QDs system is 5.119 eV (Fig. S3), which is lower than that of bulk AlN but higher than bulk GaN. This is an evidence that the embedded GaN QDs have changed the system VBM and the QCE has taken effect. Projected density of states (PDOS) of the system and different N atoms are picked out to investigate the VBM shift because the N_2*p*_ orbits mainly contribute to the VBM states (Fig. 2a). As N position shifts from AlN to GaN, and again to AlN, the VBM shifts from low to high energy, and again to low energy. The VBM of N5, which nearly locates at the center of GaN QD, is mostly close to the total VBM. It demonstrates that GaN QDs still shift up the VBM of the whole system despite strong QCE.

In the Mg-doped AlN:GaN QDs system, it is obvious that the *E*_a_ strongly depends on the doping site. We calculate the *E*_a_ of the system when Mg atoms dope at different sites. When Mg dopes at the center of GaN QD (Figs. S4a, S5a), the *E*_a_ is about 359 meV (position 1, Fig. 2b), which is quite higher than that of bulk GaN. It mainly results from the VBM down-shift induced by QCE. The increase of *E*_a_ indicates that doping in the QD is not a fine position in such system. Then Mg shifts to the interface between the AlN matrix and GaN QD (Figs. S4b, S5b), the *E*_a_ reduces to 275 meV (position 2, Fig. 2b). Although the *E*_a_ has been reduced, it is still higher than that of bulk GaN. We then dope the system with Mg atom farther away from the QD (Figs. S4c, d, S5c, d), the *E*_a_ indeed have significantly decreased just as expected, being 75 and 67 meV, respectively (positions 3 and 4, Fig. 2b). The left inset in Fig. 2b illustrates the *E*_a_ variation with doping sites. Limited by the model size, the QCE is very strong and farther doping site cannot be chosen. However, in practical doping system, the QCE can be weakened by expanding QD size and the doping site can be more far from the QD, so the *E*_a_ can be even much lower. It is worth emphasizing that it is not the farther the doping site, the higher the doping efficiency. If the acceptors are too far from the QDs to form orbital hybridization with each other, the acceptor ionization will not occur and local holes cannot freely transport in the system (right insets in Fig. 2b).

**Fig. 3:**
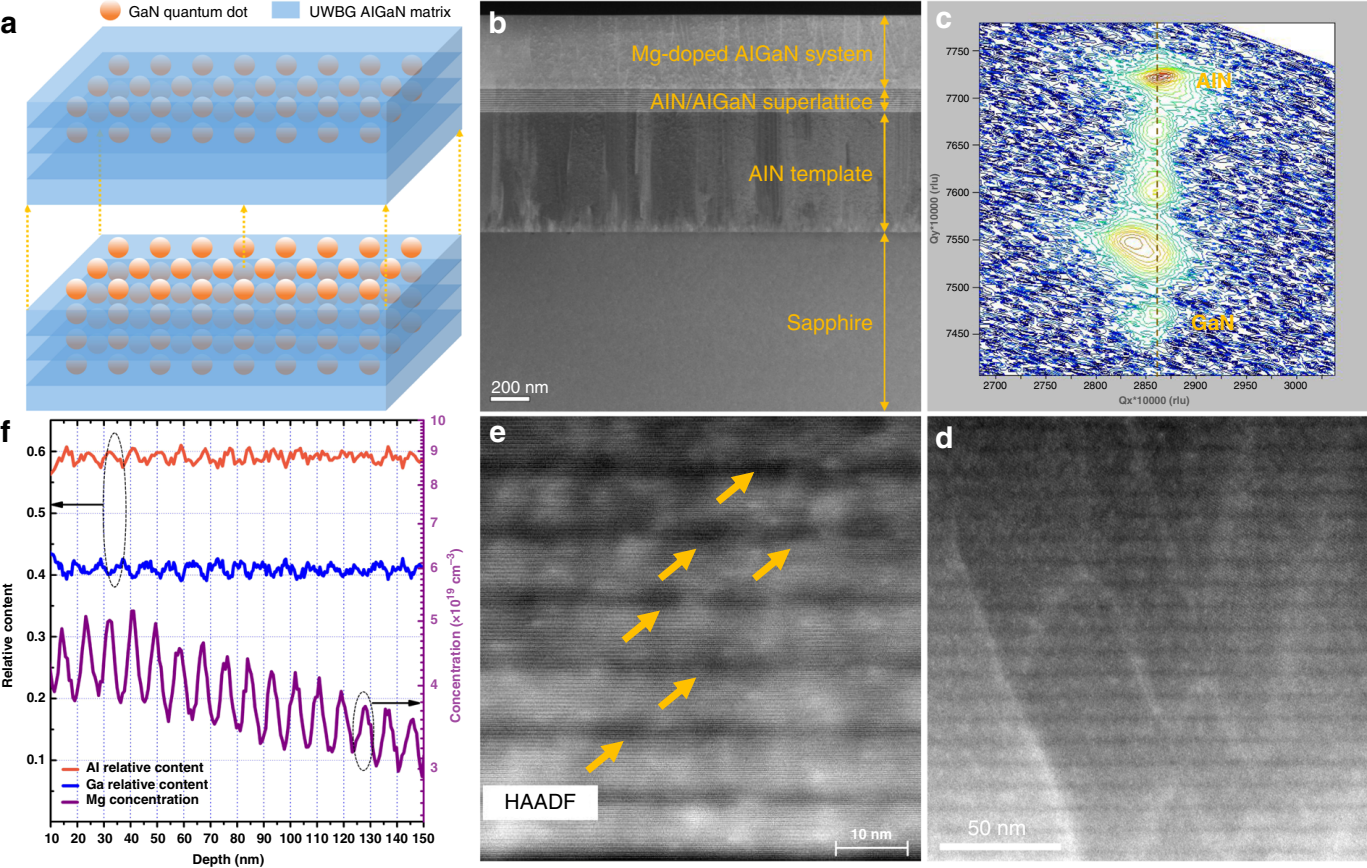
Design and characterizations of the quantum engineering Mg-doped UWBG AlGaN material. **a** Schematic diagram of the designed AlGaN:GaN QDs system. **b** Cross-sectional HAADF-STEM image of full structures of the as-grown sample. **c** XRD RSM of (−105) plane of the as-grown sample. **d**, **e** Cross-sectional HAADF-STEM images (<11–20>) of the quantum engineering Mg-doped AlGaN at different magnification. **f** SIMS of the Al, Ga, and Mg elements in the AlGaN:GaN QDs system

To experimental realize our doping conception, a Mg-doped AlGaN:GaN QDs system is designed. As we have discussed above, the key issues concerning the quantum engineering doping are the GaN QDs distributing in the AlGaN matrix and the Mg dopant concentrating in the AlGaN matrix near the interface between the AlGaN matrix and GaN QDs. Figure 3a schematically exhibits the structure design. GaN QDs are buried in the AlGaN matrix layer periodically to realize AlGaN:GaN QDs structure. The Al content of the AlGaN matrix is designed as 60%. Mg acceptors are mainly doped in the AlGaN matrix and concentrate on the hetero-structure interfaces. Under equilibrium conditions, GaN QDs in the AlGaN matrix cannot form spontaneously, and Mg dopant cannot regionally enrich spontaneously, either. Consequently, a non-equilibrium growth method is adopted. Metal-organic chemical vapor deposition (MOCVD) is applied to grow the structure. Buffer layers including AlN template and AlN/AlGaN superlattices (SLs) dislocation blocking layer are grown on c-sapphire before the AlGaN:GaN QDs system (Fig. 3b and Figs. S6, S7). Then an interrupt deposition process is applied to realize the non-equilibrium growth of the designed AlGaN:GaN QDs system (Figs. S8, S9). An AlGaN matrix layer is first grown for seconds by simultaneously injecting TMAl, TMGa, NH_3_, and Cp_2_Mg. Then the TMAl flow is stopped for seconds to grown GaN QDs. And then the TMGa flow is also stopped for seconds to form better QDs and to make the Mg atom enrich at the interface between the AlGaN matrix and GaN QDs. This procedure is repeated for 40 times and the total thickness of the AlGaN:GaN QDs system is about 370 nm (Figs. S6, S10). Finally, a thermal annealing process is implemented to remove the H atoms combined to Mg atoms (Figs. S6, S11).

X-ray diffraction (XRD) reciprocal space mapping (RSM) of (−105) and (002) planes of the as-grown sample confirms the existence of GaN and AlGaN and their periodic space distribution (Fig. 3c and Fig. S12). To further confirm the existence of periodically distributed AlGaN matrix and GaN QDs, cross-sectional scanning transmission electronic microscopy (STEM) is employed to observe the material. High-angle annular dark-field (HAADF) image obviously exhibits periodically stacked layers, indicating the existence of GaN quantum structures in the AlGaN matrix (Fig. 3d). The thickness of one period is about 10 nm and the GaN quantum structure is much thinner than the AlGaN matrix. To investigate the GaN quantum structures, higher magnification and contrast HAADF-STEM image is measured, from which the GaN quantum structures can be determined as QDs with height and diameter of about 2–3 and 6–8 nm (Fig. 3e). Contents of the Mg-doped AlGaN:GaN QDs system are determined by secondary ion mass spectrometry (SIMS) (Fig. 3f). The Al content is 60%, which agrees well with our design. The composition along the growth direction exhibits fluctuations, further indicating the existence of the GaN QDs in the AlGaN matrix. As for Mg concentration, where the Al content becomes higher, it concomitantly becomes higher, demonstrating the Mg dopants are mainly doped in the AlGaN matrix.

**Fig. 4:**
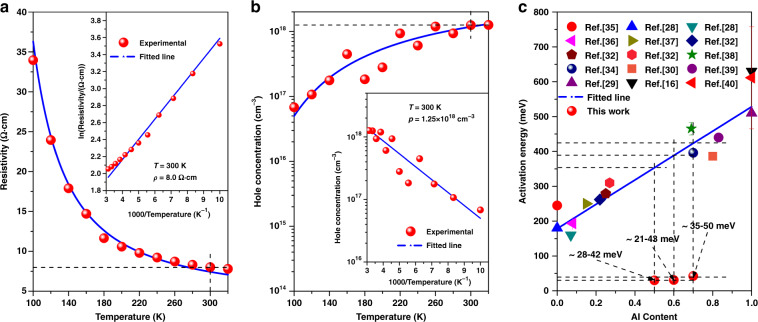
Hole conduction properties and *E*_a_ of the quantum engineering doped AlGaN. **a**, **b** Temperature-dependent resistivity and hole concentration from 100 to 320 K, respectively. The insets are the plots of reciprocal temperature (1000/*T*) verse log-scale resistivity and hole concentration. **c** Al-content-dependent *E*_a_ of Mg-doped disordered AlGaN alloys^16,28–30,32,34–40^ and the *E*_a_ of quantum engineering doped AlGaN in this work

Hall measurements are employed to test the conduction properties of the quantum engineering doped AlGaN system. The Van Der Pauw method is applied to measure the resistivity and hole concentration at different temperatures. With an increase in temperature, the resistivity exponentially decreases (Fig. 4a). The resistivity at 300 K is about 8.0 Ω cm, which is relatively lower compared to some recent results^30,31^. The *E*_a_ can be estimated by $\rho = \rho _0 \cdot {\mathrm{exp(}}\frac{{{\Delta}E}}{{k_B \cdot T}}{\mathrm{)}}$ based on the temperature-dependent resistivity, where *ρ*, *T*, *ΔE*, *k*_*B*_, and *ρ*_*0*_ are the resistivity, temperature, *E*_a_, Boltzmann constant, and fitting coefficient, respectively^32^. The fitted *E*_a_ is about 21 meV for the quantum engineering doped AlGaN system. As for the hole concentration, when temperature increases, it gradually increases due to the thermal activation of acceptors (Fig. 4b). The hole concentration at 300 K reaches the magnitude of 10^18^ cm^−3^ (1.25 × 10^18^ cm^−3^), which is a high level for disordered AlGaN alloys with Al content as high as 60%. The *E*_a_ can also be extracted by the charge neutrality condition that $\frac{{p(p + N_{\rm{D}})}}{{N_{\rm{A}} - N_{\rm{D}} - p}} = \frac{{N_{\rm{V}}}}{g}\exp ( - \frac{{{\Delta}E}}{{k_{\rm{B}} \cdot T}})$ based on the temperature-dependent hole concentration, where *p*, *N*_D_, *N*_A_, *N*_V_ and *g* stand for the hole concentration, ionized donor concentration, ionized acceptor concentration, valance band effective state density ($N_v = \frac{{2(2\pi m_h^ \ast kT)^{3/2}}}{{h^3}}$, where $m_h^ \ast$ is the hole effective mass obtained from GaN and AlN by linear interpolation method and *h* is Planck’s constant) and valance degeneracy factor (*g* = 2)^33^. The fitted *E*_a_ is about 43 meV, which is slightly higher than the temperature-dependent resistivity fitted value. Considering the size dispersion (Fig. 3e) and the mobility fluctuation with temperature which may result from the ohmic contact deterioration at low temperature (Fig. S13), we believe the actual *E*_a_ of the quantum engineering doped AlGaN system is about 21–43 meV^34^. In addition, similar *E*_a_ are also demonstrated in AlGaN with other Al contents such as 50% and 70% (Figs. S14, S15). Such an acceptor *E*_a_ is much lower than the estimated value of a disordered AlGaN alloy with similar Al content (Fig. 4c), demonstrating the effectiveness of the quantum engineering doping method.

Absorption coefficient (*α*), transmissivity (Tr), and photoluminescence (PL) spectra are measured at RT to investigate whether the quantum engineering doping method will affect the optical BE of UWBG nitrides (Figs. S16, S17). Both the *α* and Tr curves show sharp cutoff edges at solar-blind ultraviolet band and the PL spectrum also shows near-band emission at 273 nm. The inset in Fig. S16 displays the relationship between the *α* and the photon energy (*E*) near the strong absorption range, namely (*αE*)^2^ versus *E*. At strong absorption range, the curve (*αE*)^2^ versus *E* can be fitted by (*αE*)^2^ = 917.075 × (*E*-4.602). The effective band-gap of the quantum engineering doped p-type AlGaN system can be deduced to be about 4.6 eV and thus the Al content can be determined to be about 60%, which agrees well with the SIMS result. For p-type AlGaN with Al contents about 50% and 70%, the α and Tr spectra are also measured (Fig. S18). The cutoff edges are near 285 nm and 240 nm, respectively. Below the cutoff edges, both samples possess high transparency and low absorption, indicating their potential application in both near- and deep-ultraviolet optoelectronics.

**Fig. 5:**
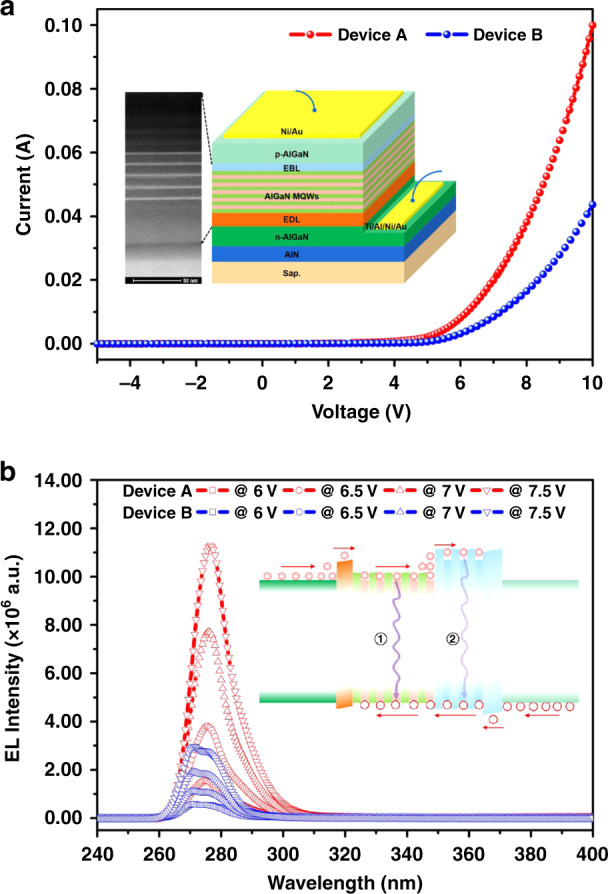
Application of the quantum engineering doping method in AlGaN-based DUV-LED. **a** *IV* curves of the devices A and B. The insets are the device structure and STEM image of the active region of the devices. **b** EL spectra of the devices A and B at different voltages. The inset denotes the energy band diagram when the devices are working

AlGaN-based deep-ultraviolet light-emitting-diode (DUV-LED) devices are fabricated to verify whether the quantum engineering doping method can benefit the device performance. AlN template is grown on sapphire before the device structure to improve the crystal quality. From bottom to up, the device structures contain n-Al_0.6_Ga_0.4_N electron transport layer, Si-doped Al_0.7_Ga_0.3_N electron deceleration layer (EDL), Al_0.6_Ga_0.4_N/Al_0.45_Ga_0.55_N MQWs active layers, Al_0.7_Ga_0.3_N/Al_0.5_Ga_0.5_N SLs electron blocking layer (EBL), p-AlGaN hole injection layer, and thin p-GaN cap layer (right inset in Fig. 5a). For comparison, the p-AlGaN hole injection layer is grown by the above-described quantum engineering doping method (Device A) and general uniform doping method (Device B), respectively. A cross-sectional STEM image of the samples illustrates that the structure agrees well with our design (left inset in Fig. 5a). Standard device fabrication processes are used to prepare the DUV-LEDs, and *IV* curves (Fig. 5a) and electroluminescence (EL) spectra (Fig. 5b) are measured to identify the device performances.

It can be seen that Device A shows lower turn-on voltage than that of Device B. At the same bias, more carriers can be injected into the active region in Device A. It indicates that the quantum engineering doped p-AlGaN hole injection layer has lower series resistance and higher hole injection efficiency than that of the general uniform p-AlGaN doped layer. EL spectra further demonstrate the superiority of the quantum engineering doping method. At the same bias, Device A possesses stronger EL intensity than that of Device B, resulting from that more holes are injected into the active region and radiative recombination rate increases. More importantly, the EL spectra of Device A show only single peak around 275 nm, while that of Device B show two peaks at about 271 and 275 nm, respectively. With the bias increasing, the EL peak of Device A gradually red-shifts from 275 to 276.5 nm, while the peaks of Devices B do not shift obviously (Fig. S19). Considering that the only different between Devices A and B is the p-AlGaN layer, the physical mechanism behind these phenomena are as follows: For Devices A, due to the better p-doping efficiency, holes can inject into AlGaN MQWs region more effectively, leading to the DUV-LED emitting at the designed wavelength of 275 nm (process ① in the inset of Fig. 5b). In addition, the lower series resistance allows the bias mainly exerting on the active region, and the quantum-confinement Stark effect results in the red-shift of the EL spectra once the bias increases. While for Device B, because of the worse p-doping efficiency, holes cannot diffuse into the AlGaN MQWs effectively and thus the electrons overflow from the AlGaN MQWs into the AlGaN SLs EBL, resulting in the unexpected DUV light emission of 271 nm (process ② in the inset of Fig. 5b). Besides, due to the higher series resistance of p-AlGaN layer of Device B, even though the bias increases, the bias exerting on the AlGaN MQWs and AlGaN SLs EBL regions is relatively low, leading to nearly no-shift of the light-emitting wavelength. The improved performance of Device A confirms the efficiency of the quantum engineering doping method.

## Discussion

The quantum engineering doping method may also work in other UWBG semiconductors such as II–VI alloys, conductive oxides, or diamond because there are abundant hetero-structures among their own congeners. In addition, the quantum engineering doping method is not limited to type-I hetero-structure like nitrides, it can also work in type-II hetero-structures. Acceptor *E*_a_ can be reduced by doping higher VBM quantum structures, while donor *E*_a_ can be reduced by doping lower CBM quantum structures. With the development of growth technology, embedding quantum structures to their UWBG semiconductor matrix counterparts can technically be realized ultimately, making the quantum engineering doping method practicable for many material systems. Furthermore, the quantum engineering doping method is not limited to UWBG semiconductors. This approach can also be attempted to apply to other conventional semiconductors once the atom-doping faces challenges, although the certain *E*_a_ value needs to be specifically determined.

In conclusion, low p-doping efficiency has long been blocking the wide applications of UWBG nitrides which possess huge potentials in next-generation semiconductor technology revolution. Lots of approaches have been developed but little progress has been made. Here, a VB modulation mode is theoretically proposed to achieve high-efficient p-type UWBG nitrides and a quantum engineering doping method is technically conducted to realize the idea, in which GaN QDs are buried in UWBG nitride matrix to produce a new BE in the system and thus to tune the system *E*_a_. First-principle calculations on Mg-doped AlN:GaN QDs system distinctly confirm the quantum engineering doping method can lower the acceptor *E*_a_ by lifting up the VBM of the whole system. By adopting non-equilibrium doping techniques, Mg-doped AlGaN:GaN QDs system is successfully fabricated. The average Al content exceeds 60% and the measured *E*_a_ is about 21–43 meV, corresponding to a hole concentration of 1.25 × 10^18^ cm^−3^ at RT. Also, similar *E*_a_ are realized in AlGaN with other Al contents of 50% and 70%. Based on the quantum engineering doping method, DUV-LEDs are also fabricated and the improved performance demonstrates the validity of the quantum engineering doping method in UWBG nitrides. It may also be applicable to other kinds of UWBG semiconductors. With the development of quantum material technologies, the quantum engineering doping method would be prevalently available and tremendously stimulate the promotion of UWBG semiconductor-based devices.

## Materials and methods

### First-principle calculation

All the density functional theory (DFT) calculations are performed by using the Vienna ab initio simulation package (VASP)^41,42^. The frozen-core projector augmented wave (PAW) approach is employed to describe the interaction between the core and valence electrons^43,44^. The generalized gradient approximation of Perdew–Burke–Ernzerhof (PBE) functional is used for the geometry relaxation^45^. The Heyd–Scuseria–Ernzerhof (HSE06) hybrid functional is used for the electronic structure calculation^46^, where the Hartree–Fock exchange fraction is set to be 0.31 according to previous theoretical work^13^. The spin polarization effect is included in all the calculations. The N *(*2*s*^2^*2p*^3^*)*, Mg *(*3*s*^2^*)*, Al *(*3*s*^2^3*p*^1^*)*, and Ga *(*4*s*^2^4*p*^1^*)* are treated as valence electrons. The energy cutoff is set to be 400 eV. The energy and force convergence criteria are 10^–5^ eV and 0.02 eV Å^−1^, respectively. Based on the above parameter setup, the calculated lattice parameters for wurtzite GaN (*a* = 3.216 Å, *c* = 5.240 Å) and AlN (*a* = 3.129 Å, *c* = 5.017 Å) are in agreement with experiment^47^.

The defect formation energy in GaN and AlN where a metal atom is replaced by Mg dopant is calculated by the following formulae (1) and (2):

1 $$E_{\rm{F}}[{\rm{Mg}}_{{\rm{metal}}}^0] = E_{{\rm{tot}}}[{\rm{Mg}}_{{\rm{metal}}}^0] - E_{{\rm{tot}}}[{\rm{bulk}}] - \mu _{{\rm{Mg}}} + \mu _{{\rm{metal}}} - E_{{\rm{corr}}}$$

2 $$E_{\rm{F}}[{\rm{Mg}}_{{\rm{metal}}}^{0/ - }] = E_{{\rm{tot}}}[{\rm{mg}}_{{\rm{metal}}}^ - ] - E_{{\rm{tot}}}[{\rm{bulk}}] - \mu _{{\rm{Mg}}} + \mu _{{\rm{metal}}} - (E_{\rm{f}} + E_{{\rm{VBM}}} + {\Delta}V)$$

where $E_{{\rm{tot}}}[{\rm{Mg}}_{{\rm{metal}}}^{0/ - }]$ is the total energy derived from a supercell calculation with one Mg dopant at charge state 0/–, *E*_tot_[bulk] is the total energy for equivalent supercell containing only pure bulk material, *μ*_Mg_ and *μ*_metal_ are the corresponding chemical potentials, *E*_f_ is the Fermi level, *E*_VBM_ is the bulk VBM energy, *E*_corr_ is the correction term for the special *k*-points, Δ*V* is the correction term that aligns the reference potential of the defect supercell and the bulk^12,48^. Large enough supercells, where a single Gamma point *k*-mesh sampling can satisfy a *k*-point interval of 0.05 × 2π Å^−1^, are chosen in the calculations, so *E*_corr_ equals zero. Besides, we choose the average core levels of the atoms far from the Mg dopant as references for the potential alignment.

The defect formation energies depend on the atomic chemical potentials. To exclude the elemental phases, it is required that *μ*_Ga_ ≤ *μ*_Ga_[bulk]=−3.61 eV, *μ*_Al_ ≤ *μ*_Al_[bulk]=−4.29 eV, *μ*_N_ ≤ *μ*_N_[N_2_] = −12.15 eV, and *μ*_Mg_ ≤ *μ*_Mg_[bulk]=−1.74 eV. To avoid the formation of competing secondary phases, the chemical potentials are further restricted as the following Eqs. (3)-(5):

3 $${\Delta}\mu _{{\rm{Ga}}} + {\Delta}\mu _{\rm{N}} \le {\Delta}H_{\rm{f}}({\rm{GaN}}) = - 1.23\,{\rm{eV}}$$

4 $${\Delta}\mu _{{\rm{Al}}} + {\Delta}\mu _{\rm{N}} \le {\Delta}H_{\rm{f}}({\rm{AlN}}) = - 3.26\,{\rm{eV}}$$

5 $$3{\Delta}\mu _{{\rm{Mg}}} + 2{\Delta}\mu _{\rm{N}} \le {\Delta}H_{\rm{f}}({\rm{mg}}_3{\rm{N}}_2) = - 4.31\,{\rm{eV}}$$

Based on the above restrictions, *μ*_Ga_ = −4.85 eV, *μ*_Al_ = −7.56 eV, *μ*_Mg_ = −3.18 eV, μ_N_ = −12.15 eV for N-rich and *μ*_Ga_ = −3.61 eV, *μ*_Al_ = −4.29 eV, *μ*_Mg_ = −2.34 eV, μ_N_ = −13.39 eV for N-lean.

The Mg dopant transition level *ε*(0/–) is defined as the Fermi level position where the two charge states 0 and –1 have the same formation energy as the following formula (6).

6 $$\varepsilon (0/ - ) = \frac{{E_{\rm{F}}[{\rm{Mg}}_{{\rm{metal}}}^ - ] - E_{\rm{F}}[{\rm{metal}}_{{\rm{metal}}}^0,E_{\rm{f}} = 0]}}{{0 - ( - 1)}} = \varepsilon ^0(0/ - ) + E_{{\rm{corr}}} - {\Delta}V$$

where $$\varepsilon ^0(0/ - ) = E_{{\rm{tot}}}[{\rm{Mg}}_{{\rm{metal}}}^ - ] - E_{{\rm{tot}}}[{\rm{Mg}}_{{\rm{metal}}}^0] - E_{{\rm{VBM}}}$$.

To calculate the Mg formation energy and transition energy in pure GaN and AlN, a 6 × 6 × 4 supercell (576 atoms, Gamma point *k*-mesh sampling) is built and the Mg dopant replaces one metal atom at the center of the supercell. To calculate the Mg transition energy in AlN:GaN QDs, an AlN cubic supercell (768 atoms, Gamma point *k*-mesh sampling) whose lattice constants are 21.68 Å, 18.77 Å and 20.07 Å along (1–100), (11–20), and (0001) directions is first built, and then the central Al atoms (a cubic cell containing 96 atoms) are replaced by Ga. It results in a stoichiometric ratio of Al_0.875_Ga_0.125_N for the whole system. For the Mg dopant, we choose four different substitution sites.

### Materials growth and annealing

The Mg-doped AlGaN:GaN QDs material is grown by MOCVD on 2 inch c-Sapphire substrate. TMAl and TMGa are used as the metal-source, NH_3_ is used as the N-source, Cp_2_Mg is used as the Mg-source for p-type doping, and H_2_ is used as the carrier gas. The Sapphire substrate is first thermally cleaned at H_2_ atmosphere to remove the surface contamination. Then an AlN nucleation layer is grown on the sapphire substrate. After the nucleation process, the temperature is ramped up for recrystallization, after which an AlN layer is grown for 15 min. Right after the 15 min growth, a low-temperature AlN interlayer is employed to improve the AlN quality. Then a high-temperature AlN epilayer is continued to grow for 40 min. The total thickness of the AlN template is measured to be 750 nm. Based on the AlN template, a periodic AlN/AlGaN SLs is grown to block the dislocations. The estimated thicknesses of the AlN barrier and AlGaN well layer are both 6 nm. When the growth of AlN/AlGaN SLs finished, an AlN cap layer with a thickness of 12 nm is grown. As all the base layers are well prepared, the Mg-doped AlGaN system in which the GaN QDs are periodically buried is grown. The AlGaN matrix layer is first grown for 20 s. Then the Al-source is closed to grow GaN QDs for 10 s. To dope the interface of the AlGaN matrix layer and GaN QDs, Ga-source is closed for 25 s and Mg-source is constant. The AlGaN matrix layer, GaN QDs, and doping period are repeated for 40 times and another AlGaN cap layer is grown for 20 s. When the growth processes finished, the temperature is reduced to 900 °C for 3 min and 865 °C for 20 min at N_2_ atmosphere to anneal the acceptors. Finally, an ex-situ annealing of 900 °C at N_2_ atmosphere for 10 min is implemented to the as-grown AlGaN to further remove H atoms combined to Mg atoms.

### Device growth and fabrication

The DUV-LED structures are grown by MOCVD on 2 inch c-sapphire substrates. The growth gases used are the same as the above mentioned. In addition, SiH_4_ is used as Si-source for n-type doping. First, AlN template of about 2 μm is grown as a buffer layer of the LED structure. Then, n-Al_0.6_Ga_0.4_N of about 1.6 μm is grown as the electron transport layer. After that, Si-doped Al_0.7_Ga_0.3_N of about 50 nm is grown as an EDL. Next, AlGaN MQWs region with 5 cycles of about 60 nm in total is grown. The thickness and Al content of barrier layers are designed to be about 9 nm and 60%, while that of the well layers are 3 nm and 50%, respectively. Then, a Mg-doped AlGaN SLs EBL of about 50 nm is grown. The Al contents of barrier and well are designed as 70 and 55%, respectively. Next, the p-AlGaN layer is grown above the EBL as the hole injection layer. For Device A, the p-AlGaN layer is grown by the quantum engineering doping method as described above. For reference Device B, the p-AlGaN layer is grown by general uniform doping. The total thickness of the p-AlGaN layer is about 370 nm for both devices. Lastly, a p-GaN cap layer of about 10 nm is grown on the p-AlGaN layer to form a better p-type ohmic contact.

Plasma-enhanced chemical vapor deposition (PECVD), standard photolithography, reactive ion etching (RIE), inductive coupling plasma (ICP) etching, electron beam evaporation, thermal evaporation, and fast thermal annealing are used to fabricate the DUV-LED devices. First, SiO_2_ mask of 500 nm is grown by PECVD and the LED pattern is transported to SiO_2_ mask by photolithography and RIE. Then, the wafers are etched by ICP of about 600 nm to the n-Al_0.6_Ga_0.4_N layer to form DUV-LED mesas. After removing the residual SiO_2_ mask, Ti 30 nm/Al 100 nm /Ni 30 nm/Au 100 nm stack metal is deposited on n-Al_0.6_Ga_0.4_N layer as n-electrode by photolithography, electron beam evaporation, and thermal deposition. The n-electrode is annealed at 600 °C at N_2_ atmosphere for 30 s to make better ohmic contact. Finally, the Ni 30 nm/Au 100 nm stack metal is deposited on the mesa as p-electrode by photolithography, electron beam evaporation, and thermal deposition. Also, the p-electrode is annealed at 550 °C at N_2_ atmosphere for 5 min to form better ohmic contact.

### Characterizations and measurements

The XRD RSM measurements are performed on a Bruker D8 Discover equipment using the Cu K_α1_ radiation line with a wavelength of 0.15406 nm at fast RSM scanning mode. The diffraction planes are (002) and (−105) planes of the samples. The SIMS measurements are applied to analyze the element distribution. The testing depth is about 600 nm beneath the surface and the testing area is 200 μm × 200 μm. The testing step length for Al, Ga, and Mg is about 1 nm and that for H is about 5 nm. The TEM-ready samples are prepared using the in situ focused ion beam (FIB) lift out technique on a FEI Dual Beam FIB/SEM. The samples are capped with sputtered C and e-Pt/I-Pt prior to milling. The TEM lamella is about 100 nm. The samples are imaged by a FEI Tecnai TF-20 FEG/TEM at 200 kV in bright-field (BF) STEM mode, dark-filed (DF) STEM mode, HR-STEM mode, and HAADF-STEM mode. The STEM probe size is 1-2 nm nominal diameter. To perform Hall measurements by Van Der Pauw method, the annealed samples are cut into 8 mm × 8 mm and Ni/Au (20 nm / 60 nm) electrodes are deposited by electron beam evaporation on four corners and annealed at 550 °C for 5 min at N_2_ atmosphere. The Hall measurements are carried from 100 to 320 K at a magnetic field of 9 kG. The α and Tr spectra are measured by UV2802S UV–VIS spectrophotometer at RT. A contrast sample that contains all structures except for the Mg-doped AlGaN epilayer is used to exclude the influences of other layers on the measurements. The *IV* curves of the DUV-LED devices are measured by Keithley 2400 and the EL spectra are measured using Omni-λ 300i grating spectrometer with 1200 line/mm at RT. The wavelength step is fixed at 0.5 nm.

## Supplementary information

Supplementary information
