# Supplementary material for: Quantum engineering of non-equilibrium efficient p-doping in ultra-wide band-gap nitrides
Source: Light Sci Appl. 2021 Mar 31;10:69. doi: 10.1038/s41377-021-00503-y (PMC8012702; doi:10.1038/s41377-021-00503-y)
Supplement: Supplementary file 1 — Supplementary information [file 41377_2021_503_MOESM1_ESM.pdf]

Supplementary Information For

Quantum Engineering of Non-equilibrium Efficient *p*-Doping in Ultra-wide Band-gap Nitrides

Ke Jiang,[1,2] Xiaojuan Sun,[1,2] Zhiming Shi,[1,2] Hang Zang,[1,2] Jianwei Ben,[1,2] Hui-Xiong Deng,[2,3]* and Dabing Li[1,2]*

1. State Key Laboratory of Luminescence and Applications, Changchun Institute of Optics, Fine Mechanics and Physics, Chinese Academy of Sciences, Dongnanhu Road No. 3888, Changchun 130033, China

2. Center of Materials Science and Optoelectronics Engineering, University of Chinese Academy of Sciences, Yuquan Road No. 19, Beijing 100049 , China

3. State Key Laboratory of Superlattices and Microstructures, Institute of Semiconductors, Chinese Academy of Sciences, Qinghuadong Road No. 35, Beijing 100083, China

*Corresponding Authors:

E-mail: hxdeng@semi.ac.cn; Phone number: +86-10-82304326 (Hui-Xiong Deng).

E-mail: lidb@ciomp.ac.cn; Phone number: +86-431-86176345 (Dabing Li).

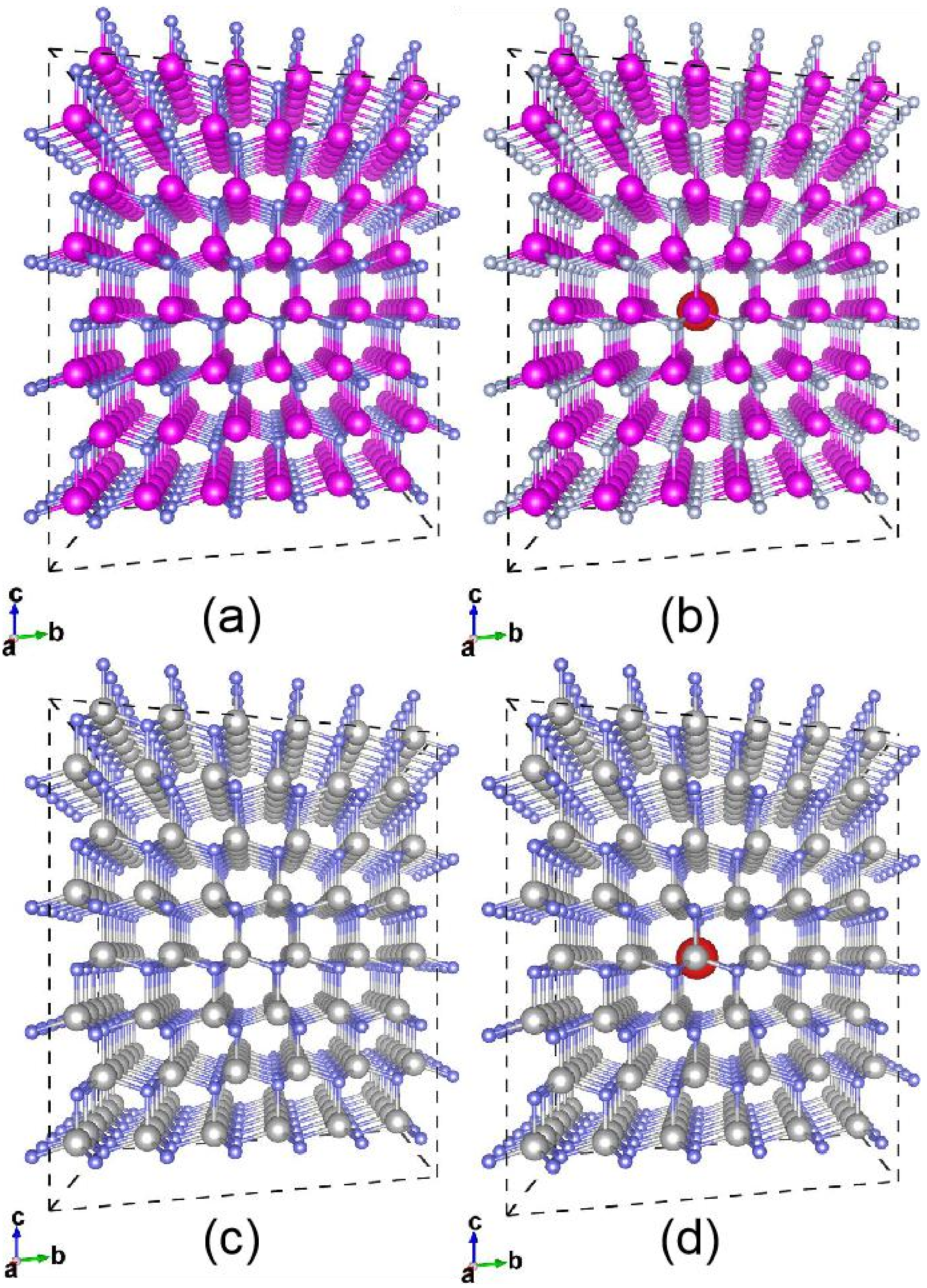

FIG. S1. Configurations of (a) bulk *GaN*, (b) bulk *GaN:Mg*, (c) bulk *AlN*, and (d) bulk *AlN:Mg* used in the first principle calculations.

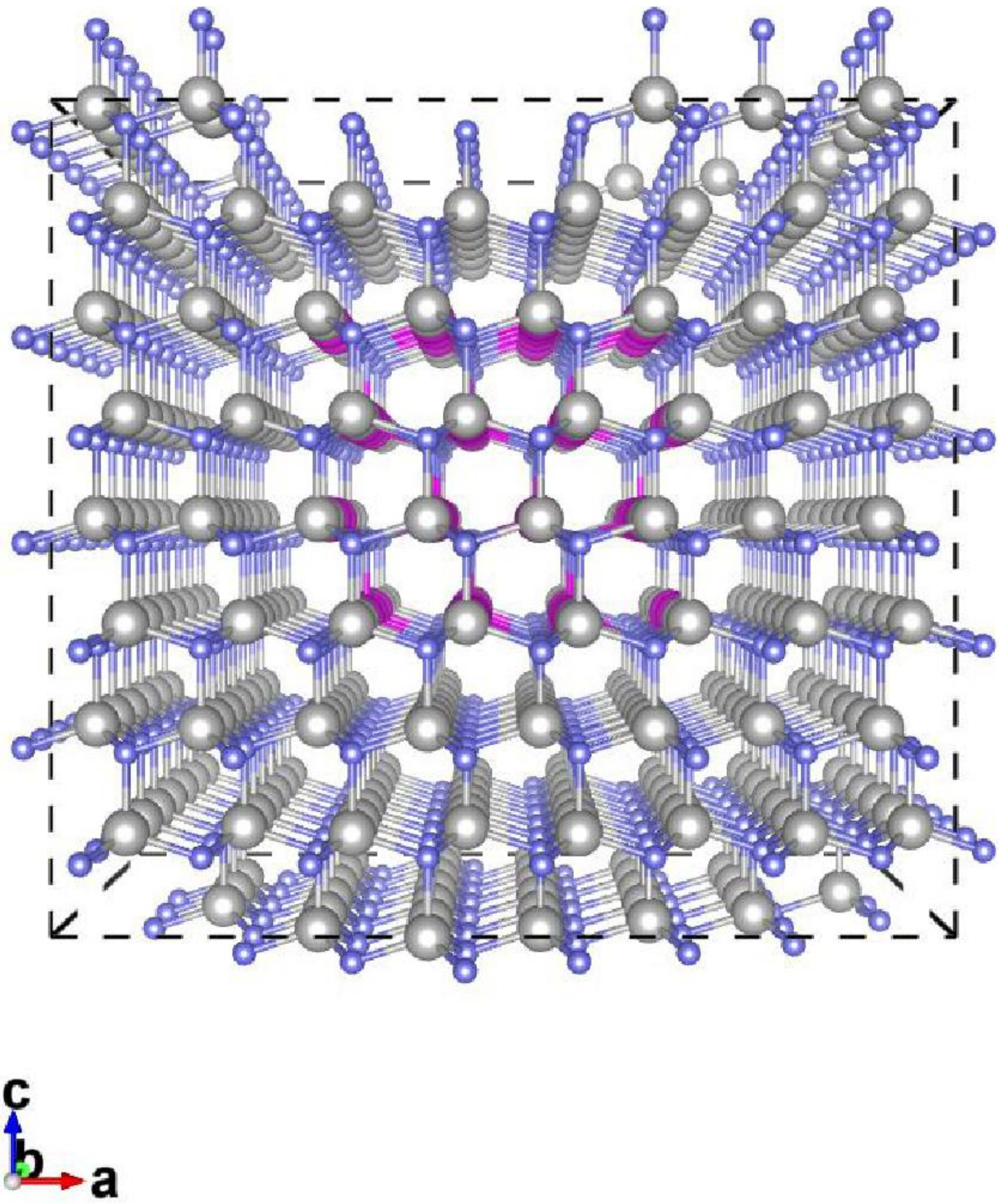

FIG. S2. Configuration of the quantum engineering doped system *AlN:GaN* QDs used in the first principle calculations.

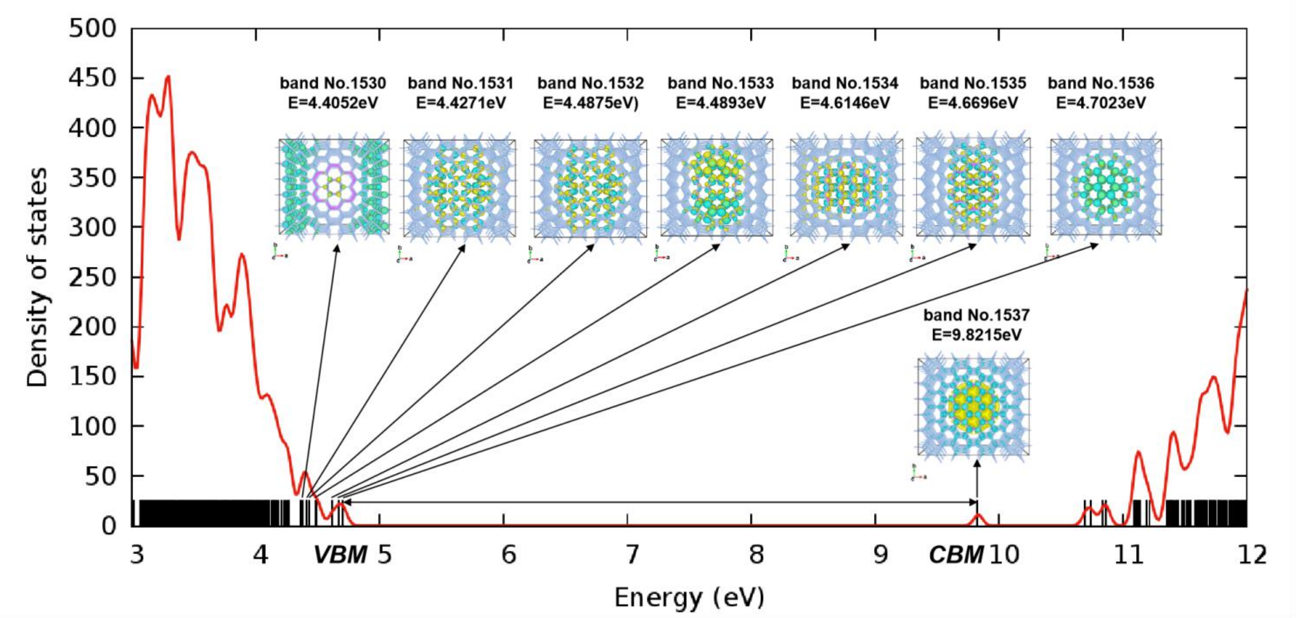

FIG. S3. The density of states of the *AlN:GaN* QD system. The energy levels of *VBM* and *CBM* are 4.7023 and 9.8215 eV, and thus the effective band-gap is 5.119 eV as calculated. The insets are the real part of the wave-functions of different levels in valance band and conduction band. From the wave-functions, it can be clearly seen that the highest occupied band (*VBM*) and lowest occupied band (*CBM*) are both contributed by *GaN* QD. In addition, the highest occupied band contributed by *AlN* matrix lies at band No. 1530, which is far from the *VBM*, demonstrating the *VBM* of the system is mainly determined by the buried *GaN* QD.

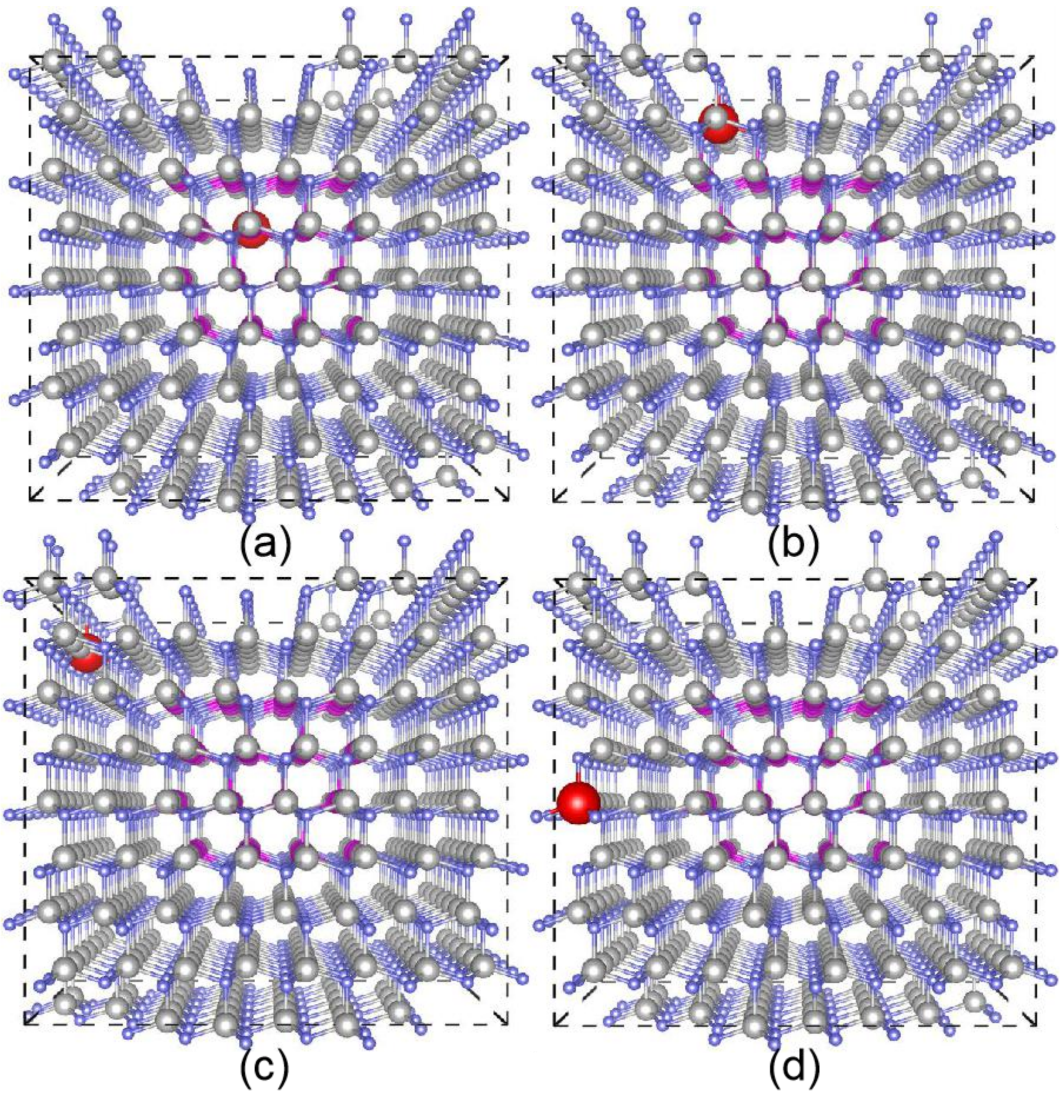

FIG. S4. Configurations of *Mg*-doped *AlN:GaN* QDs used in the first principle calculations. *Mg* atom is at (a) position 1, center of *GaN* QD, (b) position 2, interface between *AlN* matrix and *GaN* QD, (c) position 3, near the interface but further away from *GaN* QD, and (d) position 4, near the interface but further than position 3 away from *GaN* QD.

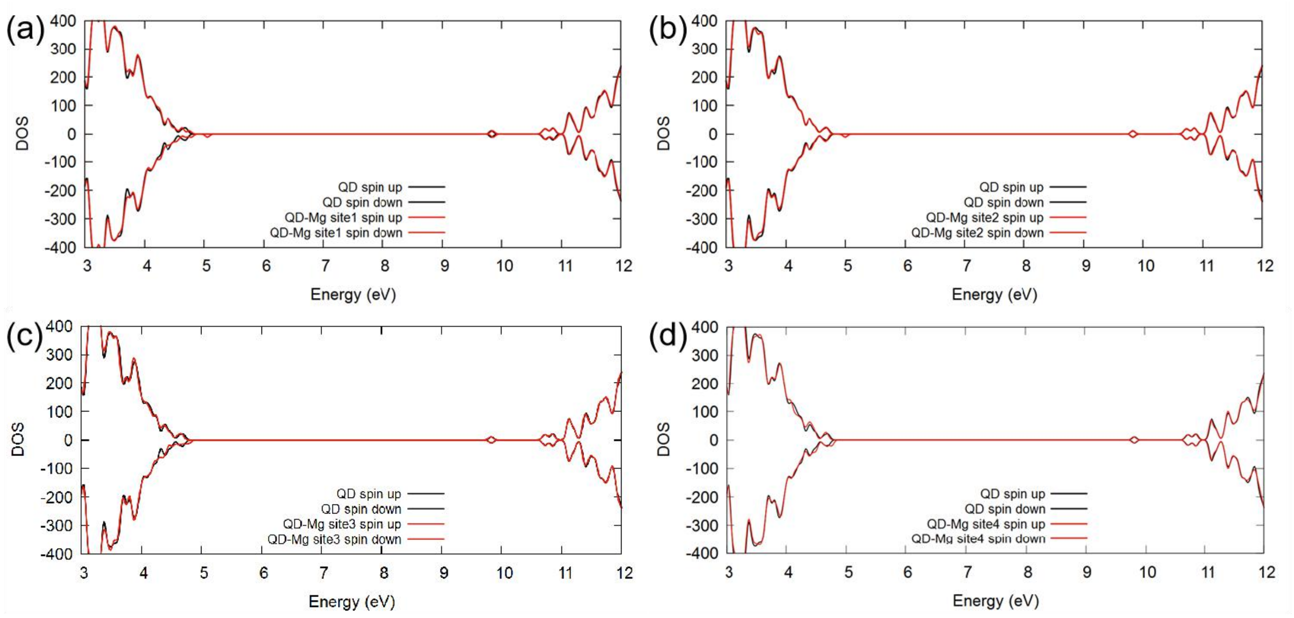

FIG. S5. The calculated density of states of *AlN:GaN* QDs and *Mg*-doped *AlN:GaN* QDs at (a) position 1, (b) position 2, (c) position 3, and (d) position 4. The black curves belong to *AlN:GaN* QDs and the red ones belong to *Mg*-doped *AlN:GaN* QDs. As is seen, the *Mg* dopant induces an acceptor level near the *VBM*. With the doping position changing from 1 to 4, the acceptor level gets closer to the *VBM*, resulting lower acceptor activation energy.

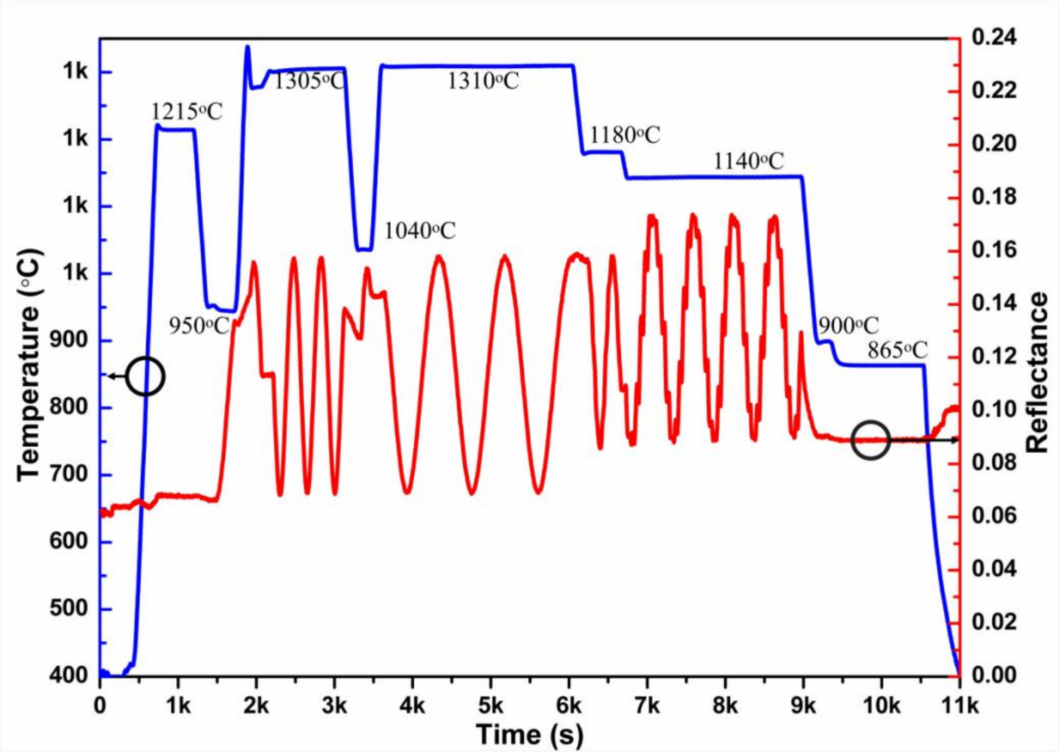

FIG. S6. The temperature and the reflectance curves during the wafer growth. As the growth proceeding, the reflectance curve oscillates steadily. It demonstrates the material is grown as designed The after-annealing is proceeded when the material growth is finished to preliminarily remove the *H* atoms connected to *Mg* atoms. Besides, there are some burrs on the reflectance curve when *AlGaN* is grown. These burrs occur when the metal organic source is stopped, indicating the surface of the wafer becomes rougher. This phenomenon may imply the formation of *GaN* QDs to some degree.

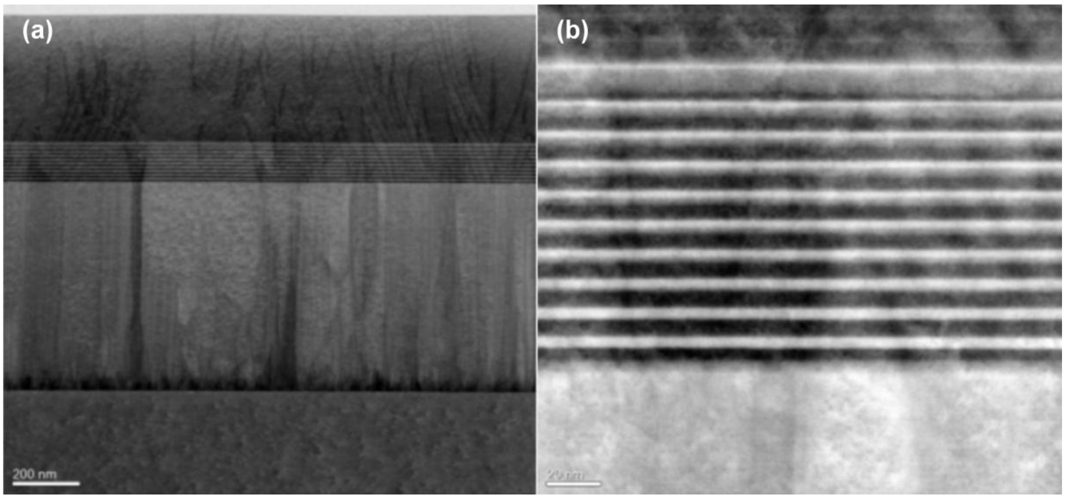

FIG. S7. TEM of the as-grown wafer. (a) The BF-STEM image of the whole structure. It shows that there are many dislocations vertically penetrating through AlN template. These dislocations are mainly formed due to grain coalescence. (b) The HAADF-STEM image of the *AlN/AlGaN* SLs. The *AlN/AlGaN* SLs between the *AlN* template and the *p-AlGaN* system is mainly designed to block the dislocations in the *AlN* template to penetrate into the *p-AlGaN* system and decrease the dislocation density. When the dislocations pass through the SLs, some disappear and some bend, resulting in dislocation density reduction.

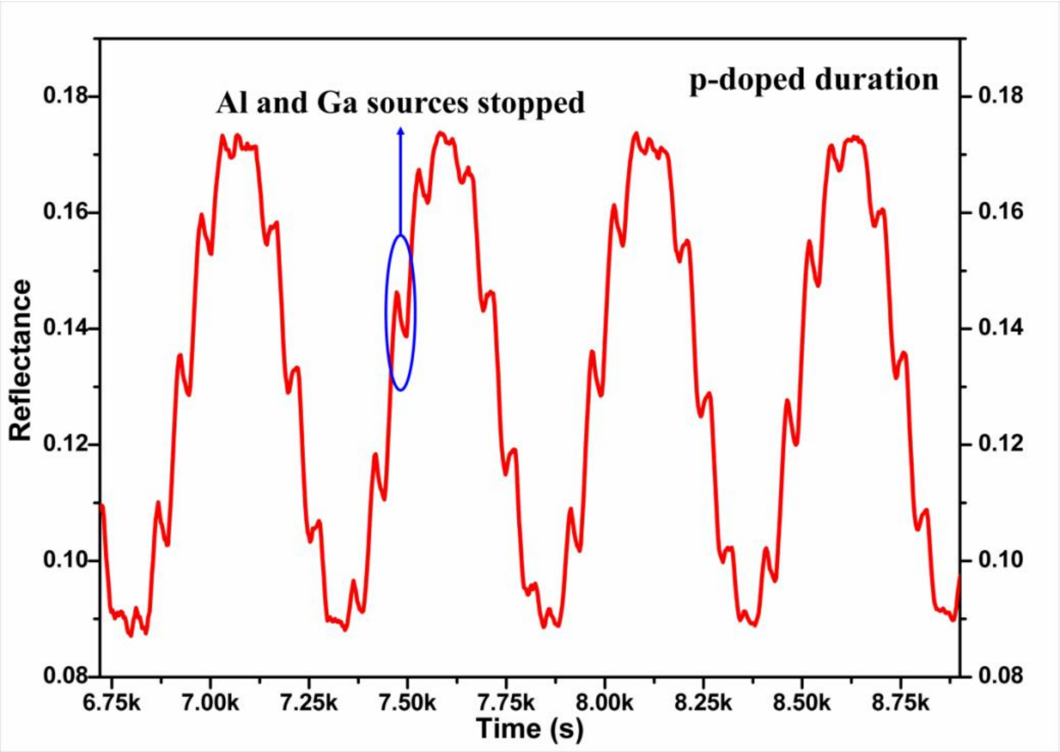

FIG. S8. The reflectance curve during the period when the *Mg*-doped *AlGaN:GaN* QDs material is grown. The overall waveform keep stable. When the *Al*-source and *Ga*-source stop, the burrs occur. The interval of these burrs is almost unchangeable. It can be deduced from the curve that the periodically distributed *AlGaN/GaN* structure is formed and *GaN* QDs are buried in the *AlGaN* matrix.

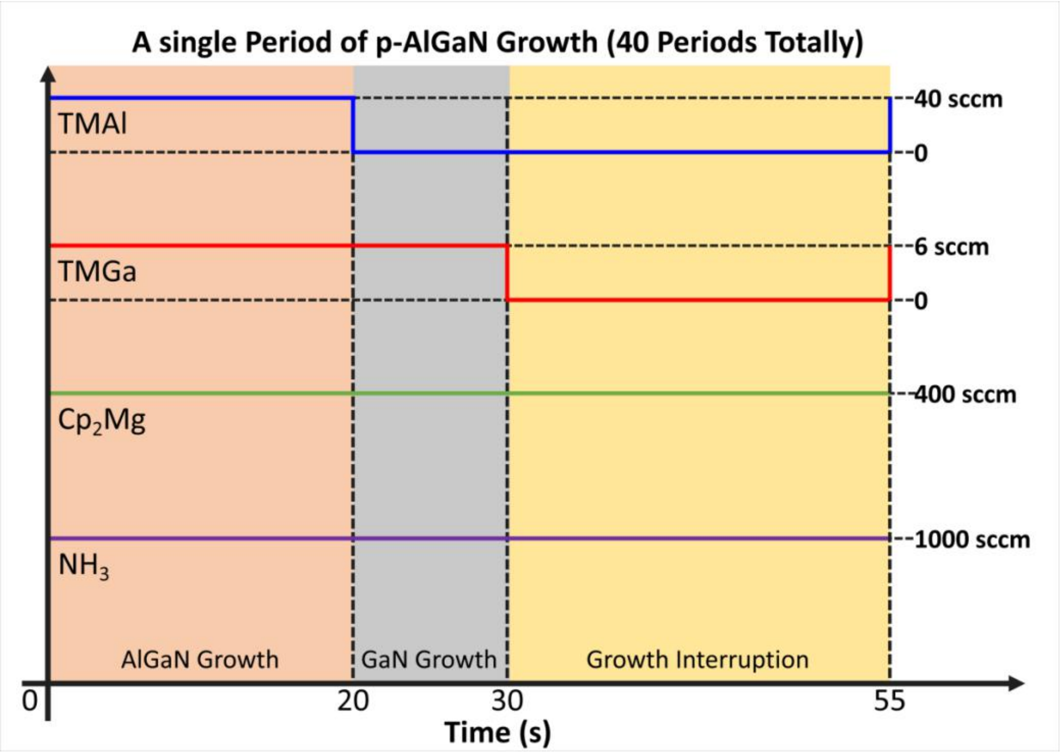

FIG. S9. The flow rate of the precursors and their on/off states within one single period *Mg*-doped *AlGaN:GaN* QDs growth. One single growth period during the *p-AlGaN* growth is 55 seconds. Within one single period, the NH_3 and Cp_2Mg are always constant. The *TMAl* source keeps open for 20 seconds, and *TMGa* source for 30 seconds. There are 25 seconds during which both *Ga*- and *Al*-source are closed. The flow rates for NH_3, Cp_2Mg, *TMAl* and *TMGa* are 1000 sccm, 400 sccm, 40 sccm and 6 sccm, respectively.

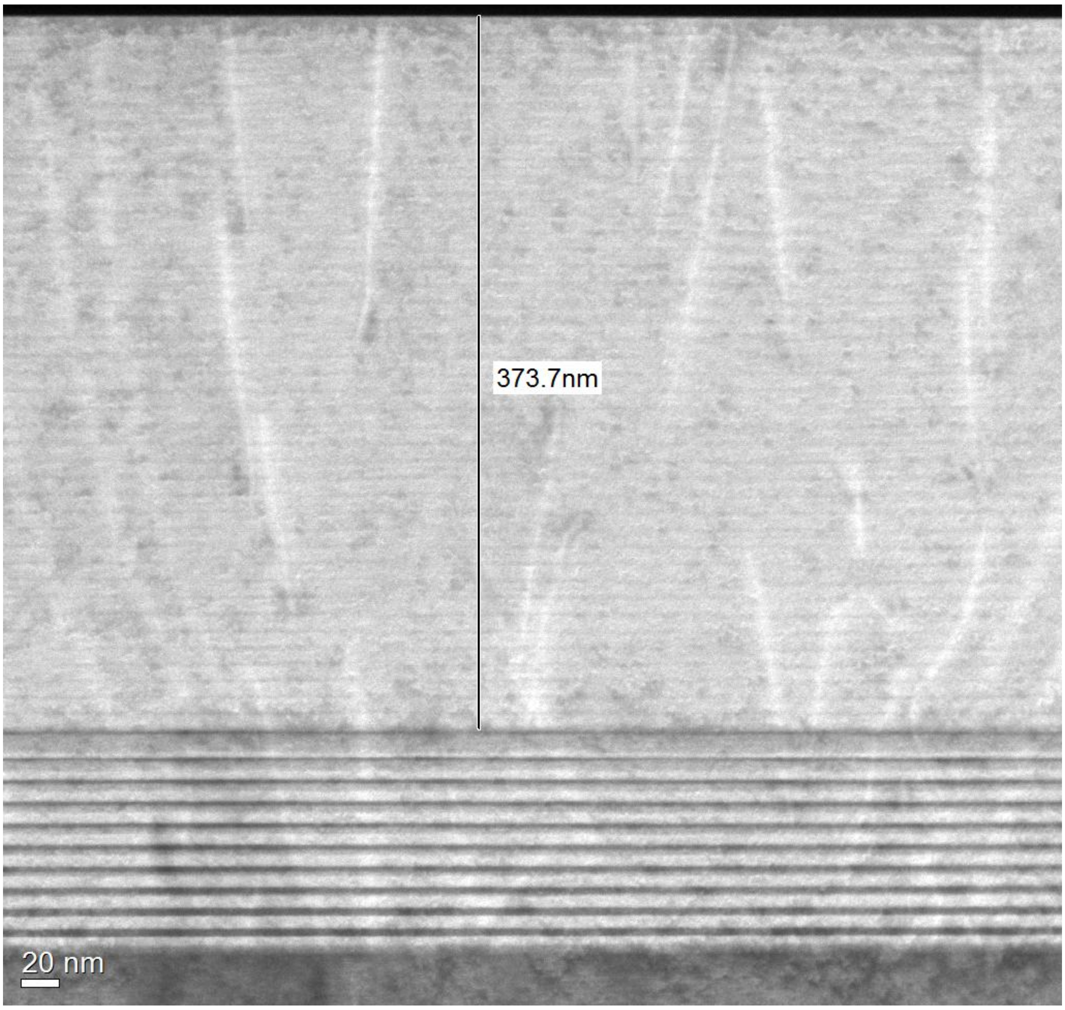

FIG. S10. The HAADF-STEM image of the *Mg*-doped *AlGaN:GaN* QDs epilayer.

The thickness of the *p-AlGaN* system is about 370 nm.

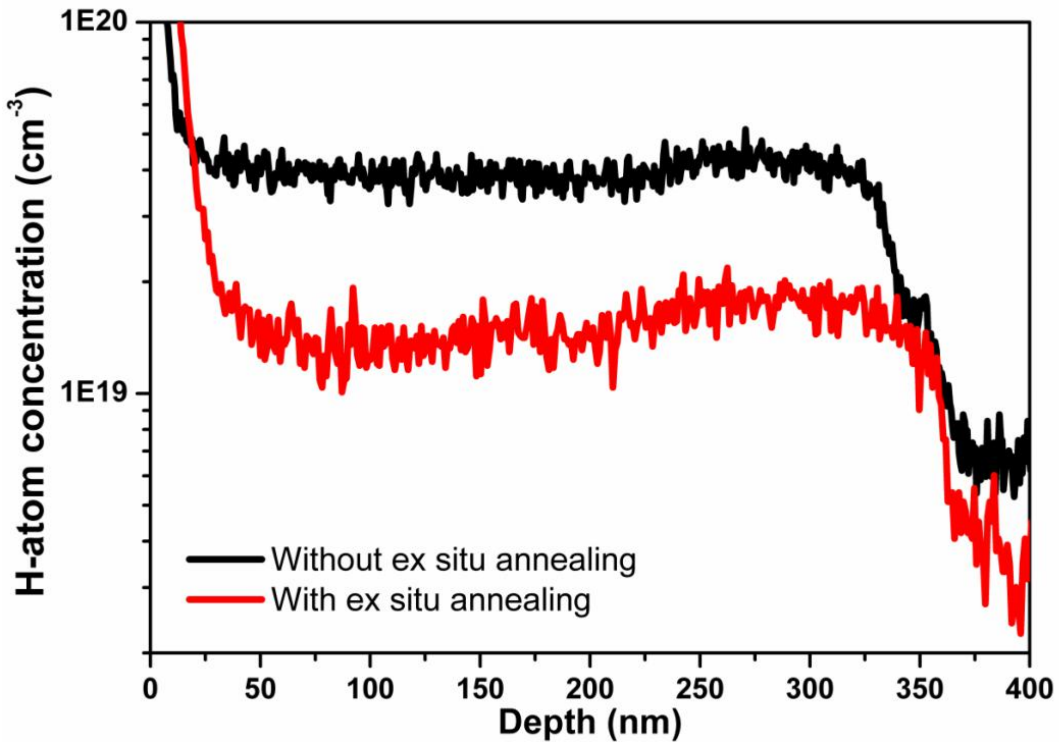

FIG. S11. The *H*-atom concentration of the *Mg*-doped *AlGaN:GaN* QDs system with and without ex-situ annealing. Without ex-situ annealing, the *H*-atom concentration in the *Mg*-doped *AlGaN:GaN* QDs system reaches 4×10^{19} cm^{-3}. At such a *H*-atom concentration, the AlGaN system cannot exhibit high hole conductivity. However, after ex-situ annealing, the *H*-atom concentration in the *Mg*-doped *AlGaN:GaN* QDs system reduced to 1.5×10^{19} cm^{-3}. The annealed *AlGaN* system exhibits good hole conductivity at room temperature. It is mainly because the *H* atoms are connected to *Mg* atoms, which can hinder the ionization of *Mg*.

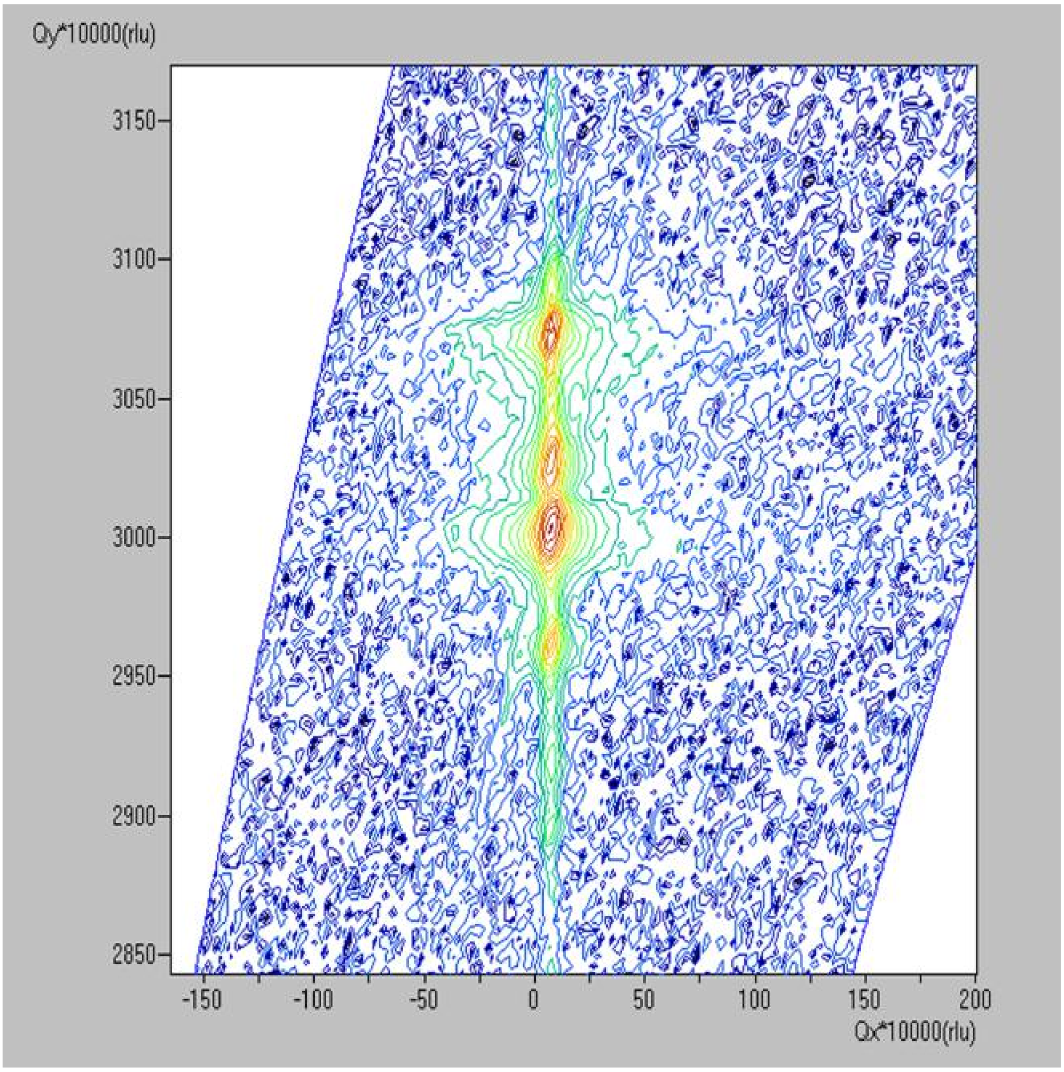

FIG. S12. The XRD RSM of the (002) plane of the *Mg*-doped *AlGaN:GaN* QDs system. It demonstrates the existence of *GaN* in the *AlGaN* system.

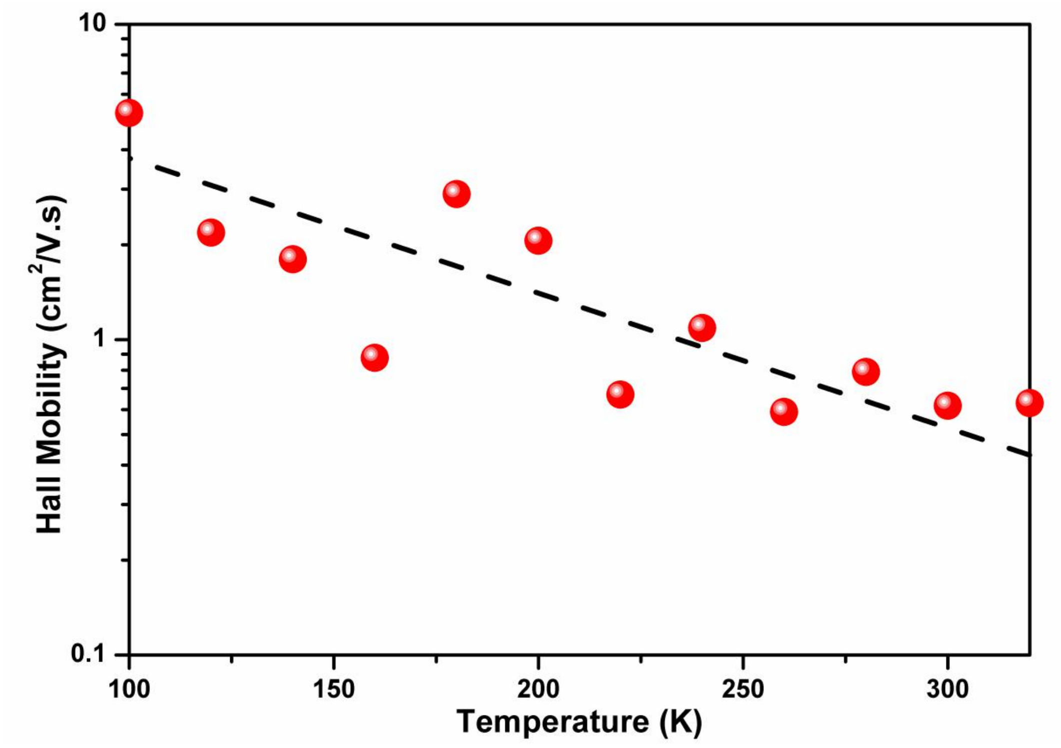

FIG. S13. Temperature-dependence hole mobility of the *Mg*-doped *AlGaN:GaN* QDs system. The dependence relationship can be expressed by the formula $\mu \propto T^{-\frac{3}{2}}$. The mobility data seems to be scattered, which is mainly due to the ohmic contact may be affect by temperature.

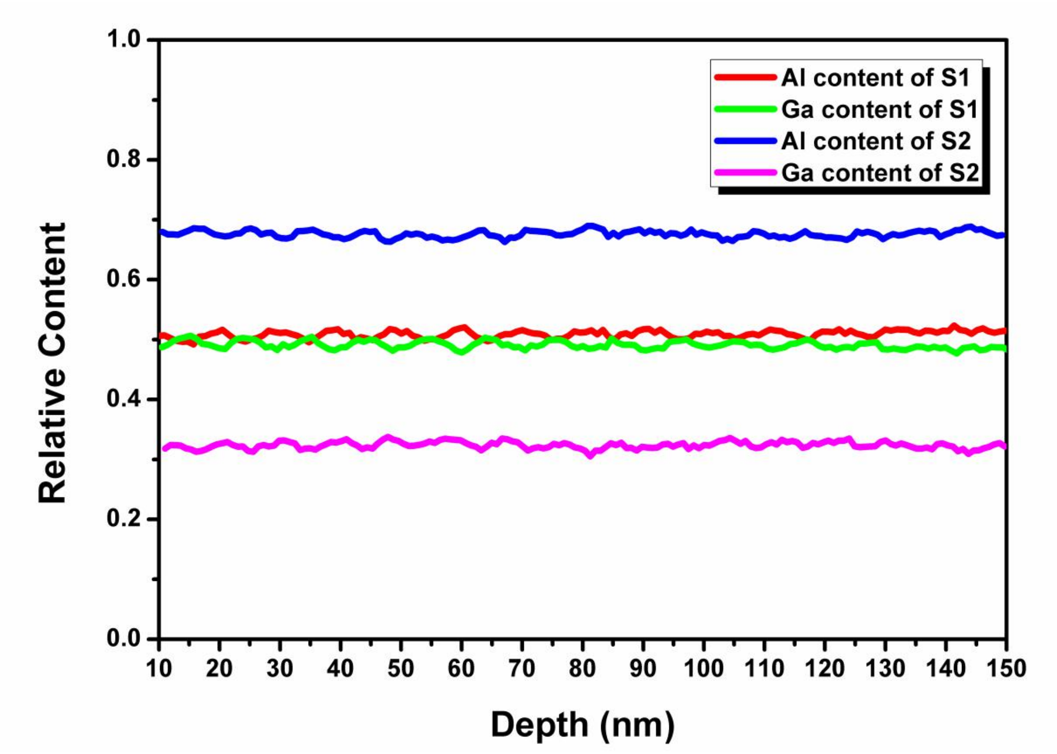

FIG. S14. The SIMS results of *Al* and *Ga* elements in *p-AlGaN* materials with other *Al* contents (labeled as Samples S1 and S2 in the figure) prepared by the quantum engineering doping method. As it can be seen, the *Al* contents are about 50% and 70%, respectively.

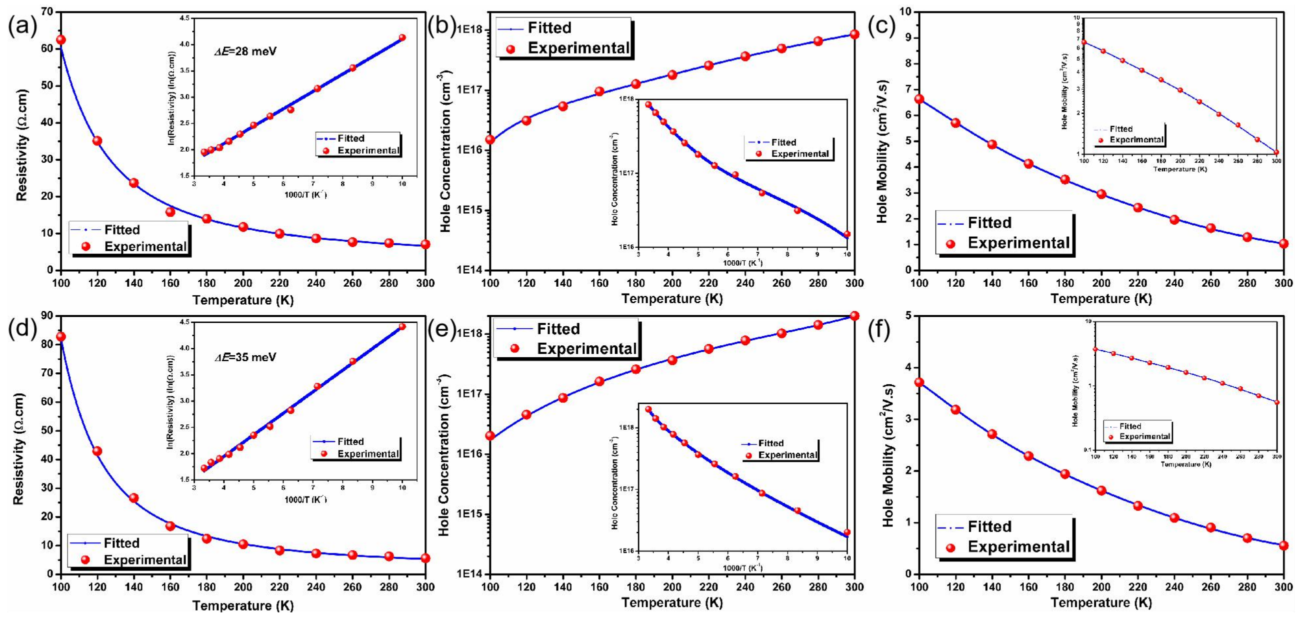

FIG. S15. Temperature dependence of (a), (d) Resistivity, (b), (e) Hole concentration and (c), (f) Mobility of *AlGaN* with different *Al* content. (a)-(c) are for *AlGaN* with *Al* content of 50% and (d)-(f) are for *AlGaN* with *Al* content of 70%. The insets are the corresponding log-scale value verse reciprocal temperature (1000/T) plots. The E_a are calculated to be 28-42 meV and 35-50 meV for $Al_{0.5}Ga_{0.5}N:Mg$ and $Al_{0.7}Ga_{0.3}N:Mg$, respectively, as described in the main manuscript. For the mobility fitting, phonon-scatterings, ionized-impurity-scatterings and neutral-impurity-scatterings are considered.

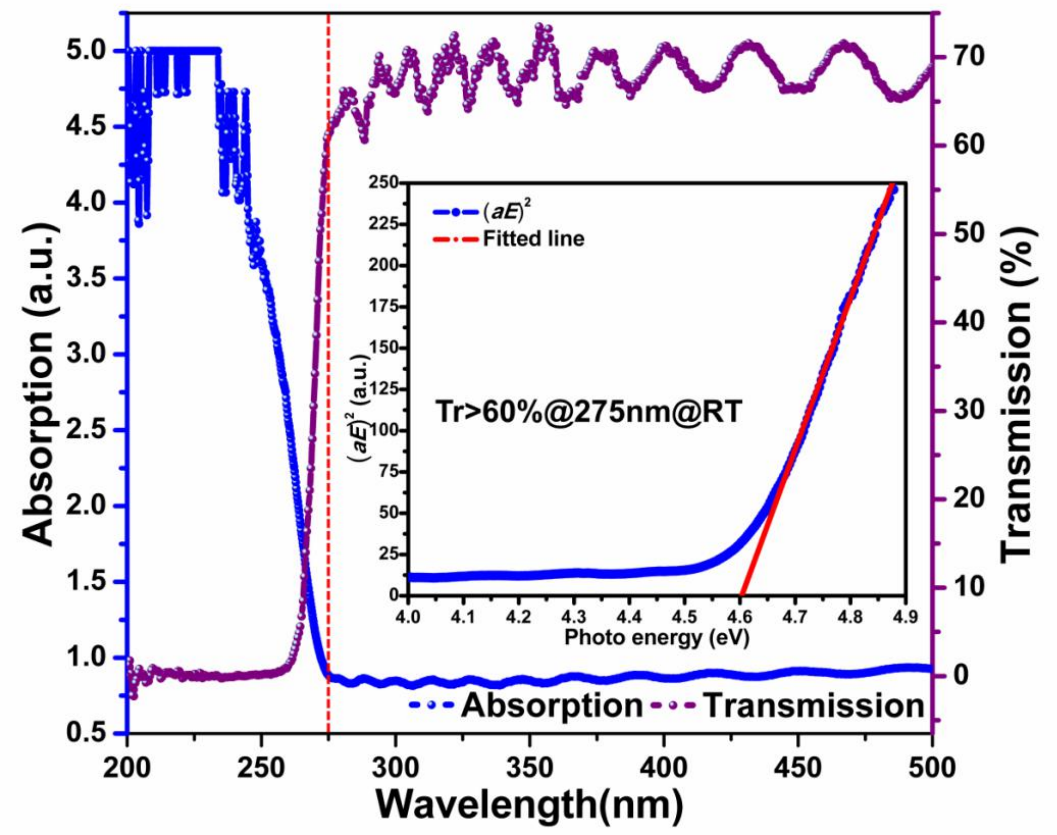

FIG. S16. The absorption and transmission spectra of the *Mg*-doped $Al_{0.6}Ga_{0.4}N:GaN$ QDs system. The insert is the dependence of $(\alpha E)^2$ on photo energy E.

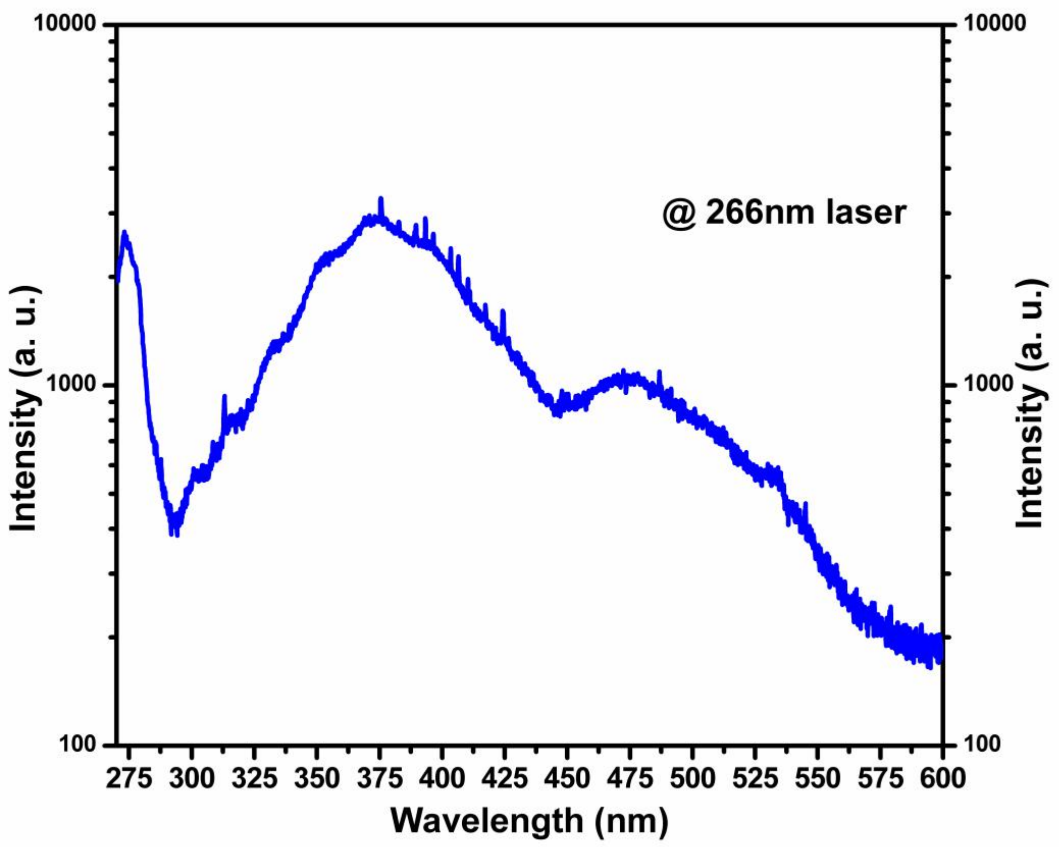

FIG. S17. PL spectrum of the *Mg*-doped $Al_{0.6}Ga_{0.4}N:GaN$ QDs system. The spectrum is excited by a laser with wavelength of 266 nm and output power of 60 mW. It exhibits three main peaks located at near 273 nm, 370 nm and 475 nm. The emission at 273 nm and 370 nm corresponds to the *AlGaN* matrix and *GaN* QDs, respectively. The emission at 475 nm is related to the *Mg* dopant level.

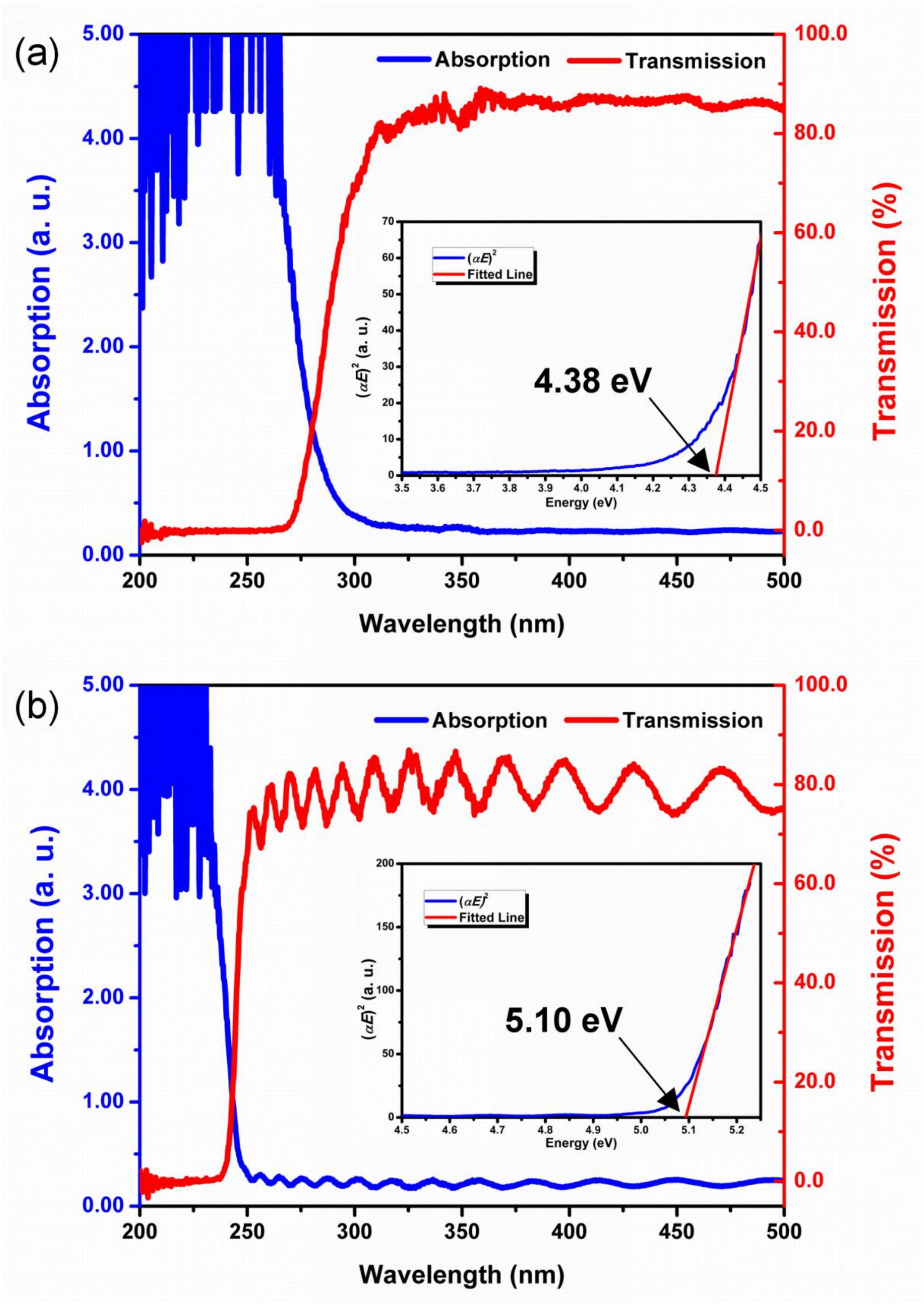

FIG. S18. The absorption and transmission spectra of *Mg-doped* (a) $Al_{0.5}Ga_{0.5}N{:}GaN$ QDs and (b) $Al_{0.7}Ga_{0.3}N{:}GaN$ QD systems. The insets are the dependence of $(\alpha E)^2$ on photo energy E. The fitted effective band-gaps are about 4.38 and 5.10 eV, respectively.

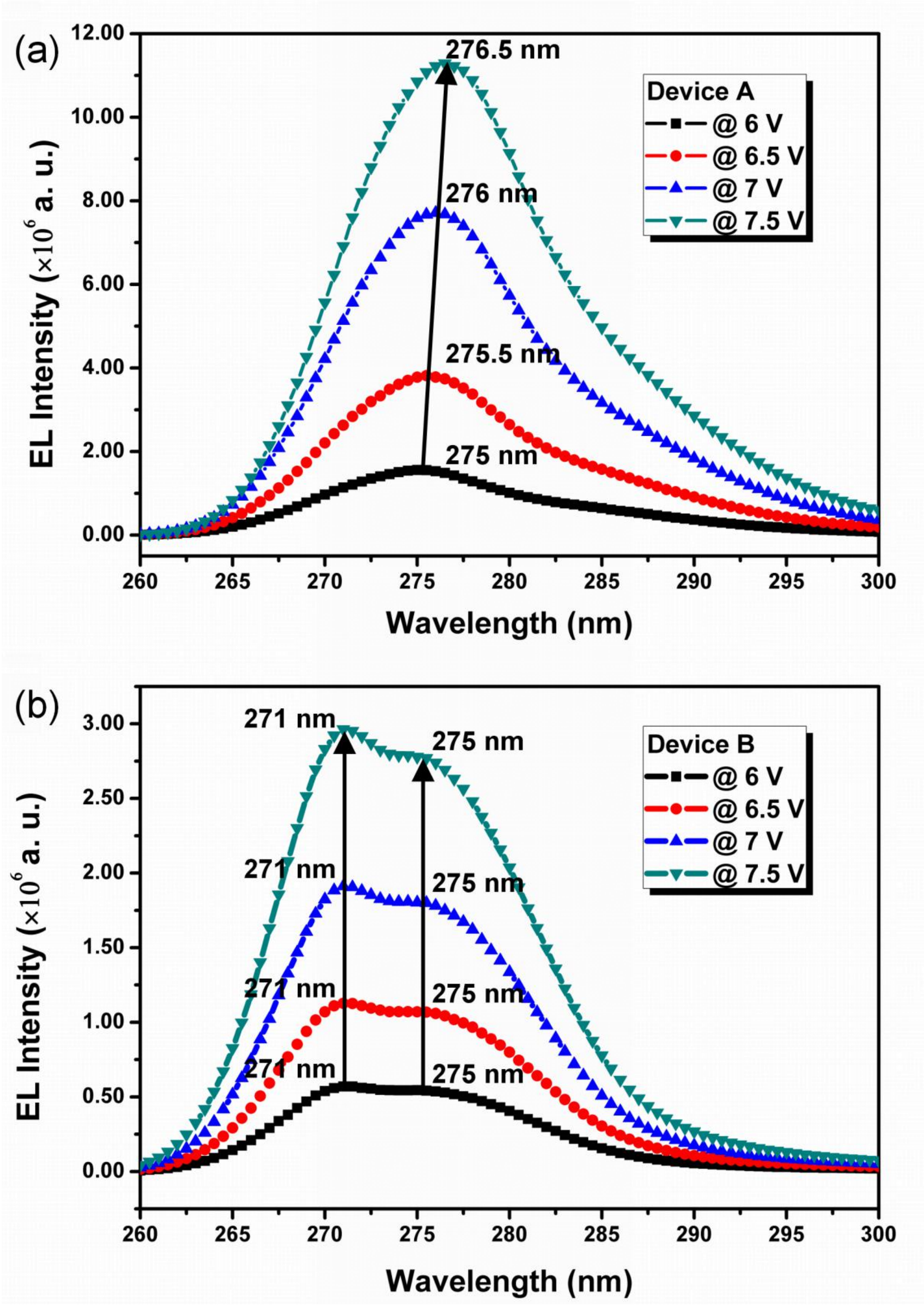

FIG. S19. Enlarged EL spectra of device (a) A and (b) B at different bias ranging from 260 to 300 nm. Device A possesses single peak at 275 nm and shows slight peak shift from 275 to 276.5 nm with bias from 6 to 7.5 V. Device B has two peaks located at 271 and 275 nm. With bias ranging, the two peak positions do not shift.
